# Ionic Liquid-Mediated Transdermal Delivery of Organogel Containing Cyclosporine A for the Effective Treatment of Psoriasis

**DOI:** 10.1021/acsomega.4c05346

**Published:** 2024-09-25

**Authors:** Deepanjan Datta, Sony Priyanka Bandi, Venkata Vamsi Krishna Venuganti

**Affiliations:** † Department of Pharmacy, Birla Institute of Technology and Science (BITS) Pilani, Hyderabad Campus, Hyderabad, Telangana State 500078, India; ‡ Department of Pharmaceutics, Manipal College of Pharmaceutical Sciences, Manipal Academy of Higher Education, Manipal, Karnataka State 576104, India; § Loka Laboratories Private Limited, Technology Business Incubator, BITS Pilani Hyderabad Campus, Jawahar Nagar, Medchal, Telangana 500078, India

## Abstract

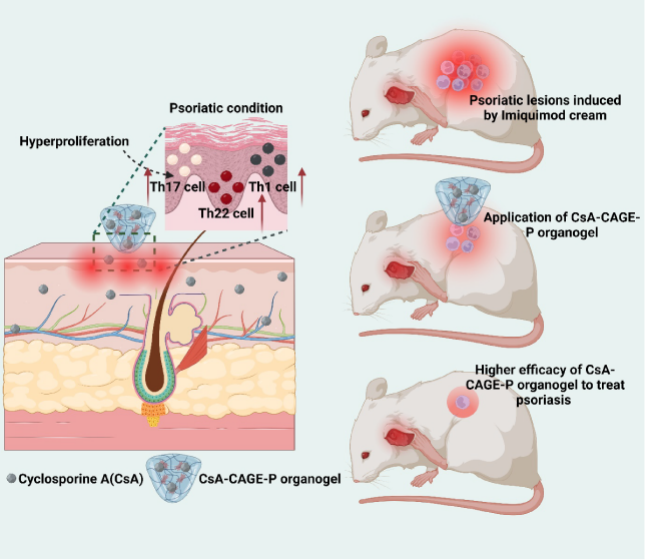

The dermal delivery of peptide therapeutics that are of high molecular weight is a challenge. Cyclosporine A (CsA) is a cyclic undecapeptide with poor aqueous solubility and high molecular weight (1202 Da) indicated for psoriasis. The objective of the study was to evaluate the effect of ionic liquids mixed with the Pluronic F127 matrix in skin permeation of CsA and its efficacy in psoriasis treatment. Choline and geranic acid (CAGE) ionic liquids in a 1:2 molar ratio were mixed with Pluronic F127 (22.7%) and PEG 400 (45%) to prepare an organogel formulation. The CsA-loaded CAGE (CsA-CAGE) and CAGE-Pluronic F127 gels (CsA-CAGE-P gel) were characterized for physical and rheological characteristics. The skin transport studies showed that free CsA did not permeate across the excised porcine skin after 48 h. The amount of CsA permeated across the oleic acid (0.25% v/v) and palmitic acid (0.25% w/v) cotreated skin was found to be 244 ± 4 and 1236 ± 17 μg/cm^2^, respectively. The application of CsA-CAGE and CsA-CAGE-P gel enhanced CsA flux by 110- and 135-fold, respectively, compared with the control. The thermal analysis and biophysical studies changed the barrier property of the skin significantly (*p* < 0.05) after incubation with CAGE and CAGE-P gel. The pharmacokinetic studies in the rat model showed that topical application of CsA-CAGE-P gel provided 2.6- and 1.9-fold greater *C*_max_ and AUC_0*–*t_, respectively, compared to the control group. *In vitro*–*in vivo* level A correlations were established with *R*^2^ values of 0.991 and 0.992 for both linear and polynomial equations for the CsA-CAGE-P gel formulation using the Wagner*–*Nelson method. The topical application of CsA-CAGE-P gel (10 mg/kg) on an imiquimod-induced plaque psoriatic model reduced the area of the psoriasis and severity index (PASI) score significantly for erythema and scaling, reversing the changes to skin thickness, blood flow rate, and transepidermal water loss. Together, CAGE-Pluronic F127 organogel was developed as an effective topical formulation for the local and systemic delivery of CsA for the treatment of psoriasis.

## 1 Introduction

Cyclosporine A (CsA) composed of 11 amino acids is a cyclic undecapeptide that acts as an immunomodulator.^1^ CsA has been indicated for treating various dermatological diseases such as atopic dermatitis, psoriasis, and alopecia areata and for systemic conditions including prevention of organ rejection after transplantation.^2^ Serious adverse effects such as chronic nephrotoxicity, renal dysfunction, and hypertension can be caused due to the long-term administration of CsA systemically.^3^ The commercially available forms of CsA are intravenous preparations, oral capsules, and eye drops.^4^ The feasibility of topical application of CsA has been explored.^5−11^ However, the permeation of CsA across the stratum corneum (SC) was hindered due to its unfavorable physicochemical properties such as molecular weight (1202 Da), log P (≈3), and poor aqueous solubility.^12^ The SC made of corneocytes and lipid matrix forms a potential barrier for the permeation of macromolecules.

Various techniques have been investigated for enhancing the permeation of impermeable active molecules through the skin.^13,14^ The techniques for enhancing skin permeation are broadly classified as chemical and physical techniques. While chemical permeation enhancers are simple systems that can be included within the topical formulations, physical permeation enhancers such as iontophoresis, microneedle devices, and ultrasound systems are complex with limitations on their clinical applicability.^15^ Chemical permeation enhancers would either improve drug diffusivity by altering the skin barrier and/or alter the thermodynamic activity of active compounds.^16,17^ To this end, alcohols, fatty acids, and terpenes are the most widely used chemical enhancers.^18−20^ More recently, ionic liquids have emerged as potential skin permeation enhancers.^21^ Here, we report the findings for the effect of fatty acids (oleic and palmitic acids) and ionic liquids (choline bicarbonate and geranic acid) on CsA penetration through the skin.

Oleic acid (OA) and palmitic acid (PA) have been found to disrupt the ordered lipid matrix within SC causing permeation enhancement.^22−24^ Ionic liquids are ionic compounds that can enhance the solubility of both polar and nonpolar compounds.^25^ The melting point of ionic liquids is below 100 °C.^26^ Among different ionic liquids, choline (C6; MW: 165.19 Da, cation) and geranic acid (C10; MW: 168.23 Da; anion) (CAGE) combination has been widely studied. The choline bicarbonate and geranic acid mixture (CAGE; 1:2 M) has been reported to enhance the permeation of proteins such as bovine serum albumin, ovalbumin, and insulin across the porcine skin.^27^Figure S1 shows the chemical structures of CsA, OA, PA, choline bicarbonate, and geranic acid.

Topical formulations include liquid and semisolid dosage forms. The semisolid preparations such as hydrogels and organogels provide ease of application. In the present study, organogel was prepared to contain a poloxamer and permeation enhancers to deliver CsA through the skin. Poloxamers are nonionic triblock copolymers of polyoxyethylene-polyoxypropylene-polyoxyethylene (PEO_*n*_-PPO_*m*_-PEO_*n*_).^28^ Pluronic F127 (also known as poloxamer 407; copolymer PEO_106_-PPO_70_-PEO_106_) is biocompatible and forms gel consistency at 37 °C.^29^ Pluronic F127 gel shows gel-to-sol transition at <25 °C.^30,31^ Pluronic F127 is commonly used as a gel matrix to prepare topical formulations containing different drugs.^32−35^

Psoriasis is a dermatological condition that has both physical and psychological effects. Psoriasis is an immuno-inflammatory disease that is caused by disruptions in the innate and adaptive immune responses in the skin.^36^ These disruptions are responsible for the development and persistence of the characteristic features of psoriasis, which include red and scaly plaques. To this end, CsA is highly successful in treating moderate to severe instances of psoriasis that require systemic therapy. However, various literature studies have been reported that have shown topical delivery of CsA for the treatment of psoriasis, using approaches including liposomes,^5^ nanocolloidal carriers,^37^ microemulsion,^38^ and niosomes,^39,40^ among others. In line with this context, our work shows the effectiveness of ionic liquids mixed with Pluronic F127 gel in enhancing the permeability of CsA into the deeper layer of the skin for the treatment of psoriasis.

## 2 Materials and Methods

### 2.1 Materials

CsA was procured from Tokyo Chemical Industry Co., Ltd., Japan. Oleic acid and palmitic acid (99+%, analytical grade) were purchased from SD Fine-Chemicals Limited (Hyderabad, India) and Sisco Research Laboratories Pvt. Ltd. (Hyderabad, India), respectively. Choline bicarbonate (technical grade, 80% in water), geranic acid (technical grade, 85% in water), acetonitrile, Kolliphor P407 oxyethylene 71.5–74.9% (Poloxamer 407), and orthophosphoric acid were procured from Sigma-Aldrich Chemical Co. (Bengaluru, India). Milli-Q (Millipore Inc., USA) water was used in all of the experiments.

### 2.2 Preparation of CsA and Permeation Enhancer Mixtures

One equivalent of choline bicarbonate (0.176 mL; 80 wt %) was added to two equivalents of geranic acid (0.408 mL; 85 wt %). The obtained mixture was subjected to stirring for 15 min at an ambient room temperature until no more carbon dioxide evolved. CsA (5.844 mg) was added to the mixture to prepare a 10 mg/mL CsA solution. The mixture was further stirred (200 rpm; 1 h) at ambient room temperature to form a clear solution.

The mixtures of CsA-OA and CsA-PA were prepared by adding 10 mg of CsA with 2.5 mg of oleic acid or palmitic acid in 1 mL of phosphate-buffered saline (PBS; pH 7.4) and isopropyl alcohol (IPA) in the ratio 65:35. For 10 min, the mixtures were vortexed. Based on solubility profiles in the 65:35 mixture of PBS and IPA, the concentration of OA and PA was chosen (Table S1).

### 2.3 Saturation Solubility Studies

The saturation solubility of CsA was studied in the vehicles, PBS + ethanol mixture, IPA, CAGE, polyethylene glycol 400 (PEG 400) + CAGE mixture, and IPA + PA mixture (Figure S2). An excess of CsA was taken, with or without the addition of chemical enhancers. The dispersions were incubated in a water bath maintained at 37 °C for 7 days. The samples were taken out at predetermined intervals and centrifuged at 10000 rpm for 10 min. The supernatant containing CsA was diluted with acetonitrile and subjected to RP-HPLC analysis. The concentration of CsA was calculated by using a calibration curve generated in acetonitrile ranging from 0.1 to 50 μg/mL, and the obtained *R*^2^ was 0.9995.

### 2.4 Preparation of CAGE-Pluronic F127 Gel

The organogel formulation was optimized by varying Pluronic F127 concentrations (4.6–22.7% w/v; Table 1). Pluronic F127 was mixed with PEG 400 at 60 °C under continuous stirring for 30 min. In a separate beaker, 5.8 mL of CAGE and CsA (58.44 mg) was mixed at room temperature for 1 h. The CAGE-CsA mixture was added to the Pluronic F127 gel base (CsA-CAGE-P gel) at 55 °C and stirred for 45 min. The resultant gel was stored at 2–8 °C until further use.

**Table 1:** Different CsA-Containing Formulations^a^

	% Weight (w/v)				
Components	F1	F2	F3	F4	F5
Pluronic 188	4.6	-	-	-	-
Pluronic 407	-	4.6	12	16.3	22.7
PEG 400	63.1	63.1	55.7	51.4	45
Choline bicarbonate	9.4	9.4	9.4	9.4	9.4
Geranic acid	21.5	21.5	21.5	21.5	21.5
Cyclosporine A	1.0	1.0	1.0	1.0	1.0
Tea tree oil	0.4	0.4	0.4	0.4	0.4

a Tea tree oil was used as a fragrance to mask the odor of formulations.

### 2.5 Characterization of CsA-Loaded CAGE-Pluronic F127 Gel

The gels were visually observed for color, clarity, and phase separation. A digital pH meter (Eutech Instruments, Hyderabad, India) was used for the pH measurement.

Rheological analysis was performed by using a modulated compact rheometer (Anton Par MCR 302, Austria). Oscillatory tests including amplitude, frequency, and temperature sweep measurements were recorded to determine the linear viscoelastic region. Amplitude sweep measurements were recorded on a 25 mm parallel plate–plate arrangement (measuring system: D-PP25; measuring cell: p-PTD200 + H-PTD120). A sample volume sufficient to spread evenly between the upper and lower plates was placed on the lower plate with a 0.5 mm measuring gap. Storage modulus, loss modulus, and complex viscosity were recorded at a constant angular frequency of 10 rad/s at room temperature while increasing the strain from 0.01% to 1000%. Then, a temperature sweep test was performed from 25 to 60 °C, at a heating rate of 2 °C/min. Viscosity was measured by varying shear rates (0.01–500 s^–1^) at 25 °C. The data were analyzed using Ostwald–de Waele or power law, calculated using eq 1:
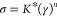
1where σ is the shear stress at break (Pa), *K* is the consistency index of fluid at a shear rate of 1 s^–1^, (Pa.s^n^), γ is the shear rate (s^–1^), and *n* is the power law index (dimensionless).

The power law model states that *n* = 1 for Newtonian samples, *n* > 1 for shear thickening (dilatant) fluid, and *n* < 1 for shear thinning (pseudoplastic) fluid.

FTIR spectroscopic analysis (Jasco FT/IR 4200, Japan) was performed for neat CsA, geranic acid, choline bicarbonate, PEG 400, Pluronic F127, blank, and CsA-loaded CAGE-P gel. For solid samples, the sample was mixed with moisture-free KBr in the ratio of 1:100 and was compressed to form a pellet. For liquid samples, the sample (20 μL) was drop-casted on the KBr pellet and was dried for 5 min. % transmittance was recorded from 4000 to 400 cm^–1^ wavenumber.

X-ray diffraction analysis (XRPD, Ultima IV, Rigaku, Japan) was performed for the neat CsA, Pluronic F127, and CsA-CAGE-P gels to ascertain their crystalline and amorphous nature. The samples were scanned from 5° to 50° 2θ using a copper Kα radiation source with a wavelength of 1.542 Å. The scanning speed and step size included 1°/min and 0.01°, respectively, at ambient temperature.

Differential scanning calorimetry (DSC, DSC-60, Shimadzu, Japan) was performed for neat CsA, Pluronic F127, blank, and CsA-loaded CAGE-P gel. Samples weighing 5–10 mg were placed in the standard aluminum pans. The thermograms were recorded after exposing the samples at a heating rate of 5 °C/min from 30 to 250 °C.

Differential scanning calorimetry and FTIR spectroscopic analysis were performed for CsA, PF127, and physical mixture (CsA + PF127) to study the drug–polymer interaction.

Porosity plays an important role in the release of the drug from the gel matrix. It is believed that an increase in the pore size or pore network increases diffusion of the drug. However, various other biological factors influence skin permeation, including skin conditions, skin age, blood flow, skin metabolism, skin hydration, temperature, pH, diffusion coefficient, drug concentration, partition coefficient, and molecular weight, among others. Nevertheless, to understand the basic drug diffusion pattern, the solvent displacement method was performed to determine the porosity. Absolute ethanol was used as a displacement solvent. CsA-loaded gel and blank CAGE-P gel were weighed accurately and immersed in absolute ethanol at different time periods. After every time point, the gel was taken out, blotted with lint-free tissue paper, and then weighed again. The change in the percent porosity with respect to time was calculated using the given formula:
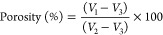
2

By suspending 0.3 g of gel in 5 mL of PBS at 37 °C, the swelling index of blank and CsA-loaded CAGE-P gel was determined in glass Petri plates. The weight of the gel was recorded before and at different time intervals during swelling experimental studies. Using eq 2, the swelling index was calculated:
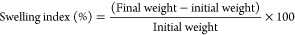
3

Scanning electron microscopy (SEM, Apreo LoVac, FEI, USA) was performed to acquire high-resolution images of the CsA-CAGE-P gel. xT Microscope Control software was used to capture SEM images at 600× magnification. Gold was sputter-coated onto the samples to a thickness of 10 nanometer layer. At a 20 kV accelerating voltage, a resolution of 1.5 nm, and a working distance of 10 mm, the images were captured.

A goniometer (Attension Theta Flex, Biolin Scientific, UK) was used to determine the contact angle of the gel placed on the excised porcine ear skin. The automatic pipette placed 10 μL of gel onto the skin’s surface using the sessile drop technique. The contact angle was measured using One Attension software at 0, 20, 40, and 60 s.

The gel sample (50 mg) was weighed, to which 10 mL of acetonitrile: water (95:5 v/v) was added. The hydroalcoholic gel solution was sonicated for 20 min to quantify the amount of CsA that was loaded in the gel. The samples were then filtered and analyzed by the HPLC method.

### 2.6 HPLC Method to Analyze CsA

CsA within the samples from *ex vivo* skin permeation experiments and *in vitro* drug release investigations was quantified using RP-HPLC (LC20-AD, Shimadzu Inc. Japan). A Luna C8 column (150 × 4.6 mm; Phenomenex Inc., USA) with a pore size of 5 μm was used. The mobile organic and aqueous phases constituted 95% v/v acetonitrile and 5% v/v deionized water, respectively. The aqueous phase contains 1% (v/v) orthophosphoric acid. The mobile phase mixtures were pumped in the ratio of 95:5 in an isocratic mode at a flow rate of 0.5 mL/min. A UV detector was used to detect CsA at a 210 nm wavelength. The standard samples were prepared in a PBS (pH 7.4)/methanol mixture (65:35) within the concentration range of 0.5–100 μg/mL. CsA elution was found to have a retention of 3.2 min (Figure S3a). The regression equation and the correlation coefficient obtained from regression analysis were *y* = 33725*x* – 41034 and 0.9999, respectively.

### 2.7 *In Vitro* CsA Release Study

*In vitro* release studies were performed using the Franz diffusion cell apparatus (PermGear Inc., USA). PBS and a methanol mixture (65:35) were used as the receptor medium. The dialysis membrane (molecular weight cut off, 2 kDa; Himedia, India) was clamped between the donor and receptor compartments. The donor compartment was loaded with 0.2 mL of CsA-CAGE or CsA-CAGE-P gel formulation. At predetermined intervals of 0.5, 1, 2, 4, 6, 8, 12, 16, 24, 36, and 48 h, samples (300 μL) were withdrawn from the sampling port and replenished with fresh media. Samples were analyzed using the above-described HPLC method. Evaluation of kinetic release data was performed using different kinetic models: zero-order, first-order, Higuchi, and Korsmeyer–Peppas by computing the obtained release data into theoretical eqs 4–7. The best-fit equation was determined based on the coefficient of determination (*R*^2^) value obtained from the plotted graph (Figure S4).
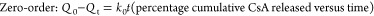
4
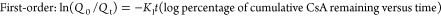
5
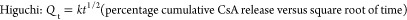
6
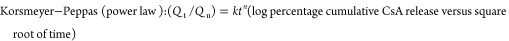
7

where *Q*_0_ and *Q*_t_ are the initial amount of CsA in organogel formulation and the amount of CsA-loaded organogel permeated. *k*_0_ and *K*_1_ are the zero-order and first-order rate constants, respectively. *K* is the constant reflecting the design variables of the system. *Q*_t_/*Q* is the fraction of CsA released over time, and “*n*” is the release exponent.

### 2.8 Skin Sample Preparation for *Ex Vivo* Studies

The freshly excised porcine ears were obtained from a local slaughterhouse. The ears were cleaned, and the dorsal skin was separated using a scalpel and forceps. A blunt scalpel was used to delicately remove the hypodermis, and the skin was examined for any obvious damage. The skin samples were stored at −20 °C for a maximum of three months.

### 2.9 TEWL and TEER Measurement

The integrity of the skin samples was studied by measuring transepidermal water loss (TEWL) and transepidermal electrical resistance (TEER).

TEWL (g/m^2^/h) was recorded by placing a vapometer (Delfin Technologies, Inc. Finland) on the donor chamber in a Franz diffusion cell. Only the skin samples with TEWL values less than 10 g/m^2^ /h were used in the study.

TEER (kΩ/cm^2^) was measured after applying a 0.3 mA/cm^2^ (I) direct current using a power supply unit. The cathode and anode were placed in the receptor chamber containing PBS–methanol (65:35) mixture and the donor chamber containing 0.2 mL of PBS, respectively. The potential difference (V) across the skin was determined using a multimeter (Fluke 189, USA). The samples with TEER values greater than 10 kΩ/cm^2^ were included in the study.

### 2.10 *Ex Vivo* Skin Permeation Studies

The CsA permeation across the intact porcine ear skin was studied by using a Franz diffusion cell setup. The skin sample was equilibrated on a Franz diffusion cell for 6 h. The skin thickness was measured using a micrometer. The thickness of the skin samples was found to be 0.35–0.45 mm. The receptor chamber was filled with 5 mL of a PBS–methanol mixture (65:35) and stirred at 600 rpm. Methanol was mixed with PBS in the receptor medium to allow sufficient dissolution of CsA and prevent saturation. The donor chamber was charged with 0.2 mL of different formulations. The effective diffusional area for the diffusion cell was 0.64 cm^2^. CsA solution (10 mg/mL) dissolved in PBS-ethanol (65:35) was used as a control formulation. The skin permeation studies were performed after cotreatment with different chemical enhancers, PA, OA, CAGE, or CAGE-P gel. The skin samples were co-treated with CsA (10 mg/mL)-CAGE, CsA (10 mg/mL)-OA, and CsA (10 mg/mL)-PA for 48 h. Similarly, CsA in CAGE-P gel at 1% and 4% (saturated condition) concentrations was applied on the intact skin for 48 h.

The sample (0.3 mL) from the receptor chamber was withdrawn at different time intervals, and the receptor media were replenished with an equal volume of PBS–methanol mixture. The RP-HPLC method described above was used to analyze the samples.

The cumulative amount of CsA permeated per unit area versus time profile was used to calculate the skin permeation parameters. The permeation parameters including flux (*J*) and the lag time (*t*_lag_) were calculated by projecting the linear component of the curve onto the time axis. The time points used for regression analysis were between 2 and 48 h. The correlation coefficient of the linear portion of the permeation profile was 0.97–0.99. The permeability coefficient (*K*_p_) and diffusion coefficient (*D*) of CsA across porcine skin were calculated by using the following equations:
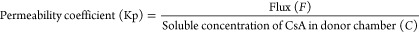
8
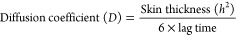
9

The skin samples were analyzed for CsA retention after 48 h. SC was removed from the viable skin by the tape-stripping technique. The treated skin area (0.64 cm^2^) was sequentially stripped five times using a Scotchbook tape (845, 3M, USA). The stripped tapes were incubated in 1 mL of PBS: methanol mixture overnight at 4 °C to extract CsA. The viable skin was cut into small pieces and homogenized using a tissue homogenizer (IKA T10 basic; Ultra-Turrax, India). The tissue sample was mixed with 500 μL of acetonitrile and sonicated for 45 min. The samples were then centrifuged at 10000 rpm for 20 min at 4 °C. The RP-HPLC procedure mentioned above was used to analyze CsA from the collected supernatant. The % recovery was estimated by comparing the peak areas of CsA extracted from the skin samples with equal known amounts of CsA.

### 2.11 Mechanistic Studies

FTIR spectroscopic studies were performed for the epidermal membranes incubated with permeation enhancers. The excised porcine ear skin was kept at 60 °C for 90 s in a water bath, followed by the separation of SC from the viable skin. The SC was treated with 0.2 mL of PA (0.25% w/v), OA (0.25% w/v), and CAGE and CAGE-P gel (0.2% w/w) at 37 °C for 12, 24, and 48 h mounted on a Franz diffusion cell. Then, the samples were washed with 3 mL of phosphate-buffered solution, blotted dry, and then air-dried for 1 h at room temperature. The dried samples were blended with potassium bromide in a ratio of 1:100. A 40 kN force was applied to compress the sample mixture into a pellet. For the control, the SC sample was treated with PBS and ethanol (65:35) mixture for similar time points. The spectra were captured using FTIR with a resolution of 4 cm^–1^ in the 4000–400 cm^–1^ wavenumber range.

For DSC, a typical 40 μL aluminum pan was filled with 2–5 mg of SC sheets. The SC samples were treated with 0.2 mL of PA (0.25% w/v), OA (0.25% w/v), and CAGE and CAGE-P gel (0.2% w/w) for 12, 24, and 48 h. A DTG-60 apparatus (Shimadzu, Japan) was used for thermal analysis. All the samples were heated from 28 to 200 °C under nitrogen flow at a 2 °C/min heating rate. The transition temperatures and enthalpy changes that correspond to them were recorded.

SEM was used to visualize high-resolution micrographs of SC samples treated with 0.2 mL of PA (0.25% w/v), OA (0.25% w/v), and CAGE and CAGE-P gel (0.2% w/w). SEM images with a magnification of 500× to 2000× were captured using the xT Microscope Control software. Gold was sputter-coated onto the SC samples to a thickness of 10 nm. The images were captured at a resolution of 1.5 nm with an accelerating voltage of 20 kV.

### 2.12 Pharmacokinetic Studies

CsA standards were prepared in rat plasma. A CsA stock solution of 1 mg/mL was prepared in acetonitrile. The plasma was spiked into the serially diluted CsA solution to achieve plasma concentrations in the range of 0.0001–18.75 μg/mL.

For test sample analysis, plasma collected from the animals was spiked with 300 μL of acetonitrile. The sample was vortexed for 15 min and centrifuged for 15 min at 11000 rpm The supernatant was collected for the quantification of CsA. For CsA analysis in the rat plasma, the RP-HPLC method was modified from the method discussed above. The flow rate was increased to 0.8 mL/min with the CsA eluted at 1.87 min. CsA was detected at a 210 nm wavelength using a UV detector. The standard calibration curve in plasma was obtained for a concentration range of 0.00037–18.75 μg/mL. The regression equation and correlation coefficient obtained were *y* = 45415*x* + 253903 and 0.9985, respectively.

The pharmacokinetic studies included male Sprague–Dawley rats that were 8 to 10 weeks old, with an average weight of 180 ± 20 g. The animal experimental protocol was approved by the Institutional Animal Ethics Committee (IAEC) of BITS Pilani, Hyderabad Campus (HYD/IAEC/2021/09). The rats were divided into four groups, with four animals in each group. The animals were anaesthetized using isoflurane and oxygen mixture in a rodent anesthesia chamber (RAS-4, EZ animal systems, Canada), using a system pressure of 30 kPa. The hair on the dorsal side was removed by using a hair clipper without causing injury. CsA formulations were applied on the dorsal skin on an area of 3 cm^2^. The formulations included CsA (40 mg) in 1 mL of PBS–ethanol mixture (0.65 + 0.35 mL, control group); CsA (40 mg) + PA (2.5 mg) solubilized in 1 mL PBS + IPA (0.65 + 0.35 mL) mixture; CsA (40 mg) in choline bicarbonate + geranic acid (0.31 + 0.69 mL) mixture; and CsA (40 mg) in choline bicarbonate + geranic acid (0.31 + 0.69 mL) dispersed in 1 g of Pluronic F127 gel base. Co-treatment of CsA with OA showed the least cumulative amount permeated in *ex vivo* studies and, therefore, was excluded from the pharmacokinetic studies. A micropipette tip was used to spread the formulations gently on the rat’s skin. Following that, 250 μL of blood was collected by puncturing the retro-orbital plexus at predefined time points of 0.5, 1, 2, 4, 8, 12, 24, 36, and 48 h in polypropylene centrifuge tubes. The blood samples were centrifuged at 7500 rpm for 15 min at 4 °C. The separated plasma samples were quantified for CsA using the HPLC method described above.

### 2.13 Development of *In Vitro*–*In Vivo* Correlation (IVIVC)

The *in vivo* absorption and *in vitro* permeability (*ex vivo* in this context) from the absorption and permeation studies across the rat and porcine skin, respectively, were utilized to develop an IVIVC model for cyclosporine-loaded CAGE-P gel. The IVIVC was created by graphing the percentage of permeation *in vitro* on the *x* axis and the comparable percentage of absorption *in vivo* on the *y* axis at the same time periods. Both linear and polynomial components with regression values (*R*^2^) were calculated to predict the *in vitro*–*in vivo* correlation. The percentage of CsA absorption (% absorption) was estimated using the Wagner*–*Nelson equation^41^ (10):
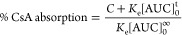
10

where *C* and *K*_e_ are the concentration and elimination rate constant, respectively, at specific time points;  is the area under the plasma concentration curve from time 0 to t; and  is the area under the plasma concentration curve from time 0 to infinity.

### 2.14 Efficacy Studies

The imiquimod (IMQ)-induced plaque psoriasis model was developed to evaluate the effectiveness of CsA-CAGE-P gel. Male SD rats aged 8 to 10 weeks and weighing 180–220 g were used for the study. The rats were divided into four groups, with four animals in each group. Group I: sham control with no psoriasis induced; group II: negative control, without treatment; group III: treated with CsA-CAGE-P gel (10 mg/kg/day); group IV: treated with CsA intravenous injection equivalent to a 10 mg/kg dose of CsA-CAGE gel formulation.

IMQ cream (5% w/w; 62.5 mg) was applied on the shaved back of rats spread on an area of 3 cm^2^ for eight consecutive days. The animals were treated with respective formulations from the 9th day to the 16th day. TEWL, LDF, changes in body weight, and food and water intake were recorded every day.

The body temperature of the rat was measured by using an infrared thermal camera (FLIR E60, USA). The thermal camera was placed at a 1 m distance from the animal and imaged with fixed parameters such as body emissivity, 0.98; relative humidity, 50%; and atmospheric temperature, 24 °C. The thermal images were analyzed using FLIR thermal studio software version 1.9.10 (Teledyne FLIR LLC, USA).

#### 2.14.1 PASI Scoring

The psoriasis area and severity index (PASI) was recorded to understand the severity of psoriasis in rats. In a blinded study, five volunteers who were not related to the study examined the rats for erythema (redness) and desquamation (scale) visually. The PASI scores were recorded on days 0, 2, 4, 8, 10, 12, 14, and 16. Each of the parameters received a score between 0 and 4 (0, none; 1, mild; 2, moderate; 3, severe; 4, very severe). The thickness of the skinfold on the back was determined using a vernier calliper.

On the 16th day, the animals were sacrificed by cervical dislocation. The dorsal skin was harvested and kept at −80 °C for further investigation. The spleen was harvested to record the spleen-to-body weight ratio.

#### 2.14.2 Histopathological Analysis

The skin sample was placed at −80 °C for 12 h to embed it in an optimum cutting temperature (PolyFreeze, Polysciences, Inc., USA) compound. The skin sample was cut into 5 μm thin sections using a cryotome (CM1520, Leica Biosystems, Germany). Hematoxylin and eosin staining was performed for histological evaluation. Microscopic images of the sections were acquired using an optical microscope (Olympus IX53, Olympus Corporation, Japan).

### 2.15 Statistical Analysis

The results of 4–5 experimental repetitions are presented as the mean ± standard deviation. The differences between various treatment groups were compared using the one-way ANOVA or unpaired *t* test (GraphPad Prism, 5.03 version). The difference between groups was considered significant when *p* < 0.05.

## 3 Results

### 3.1 Characterization of CsA-CAGE-P Gel

**Figure 1:**
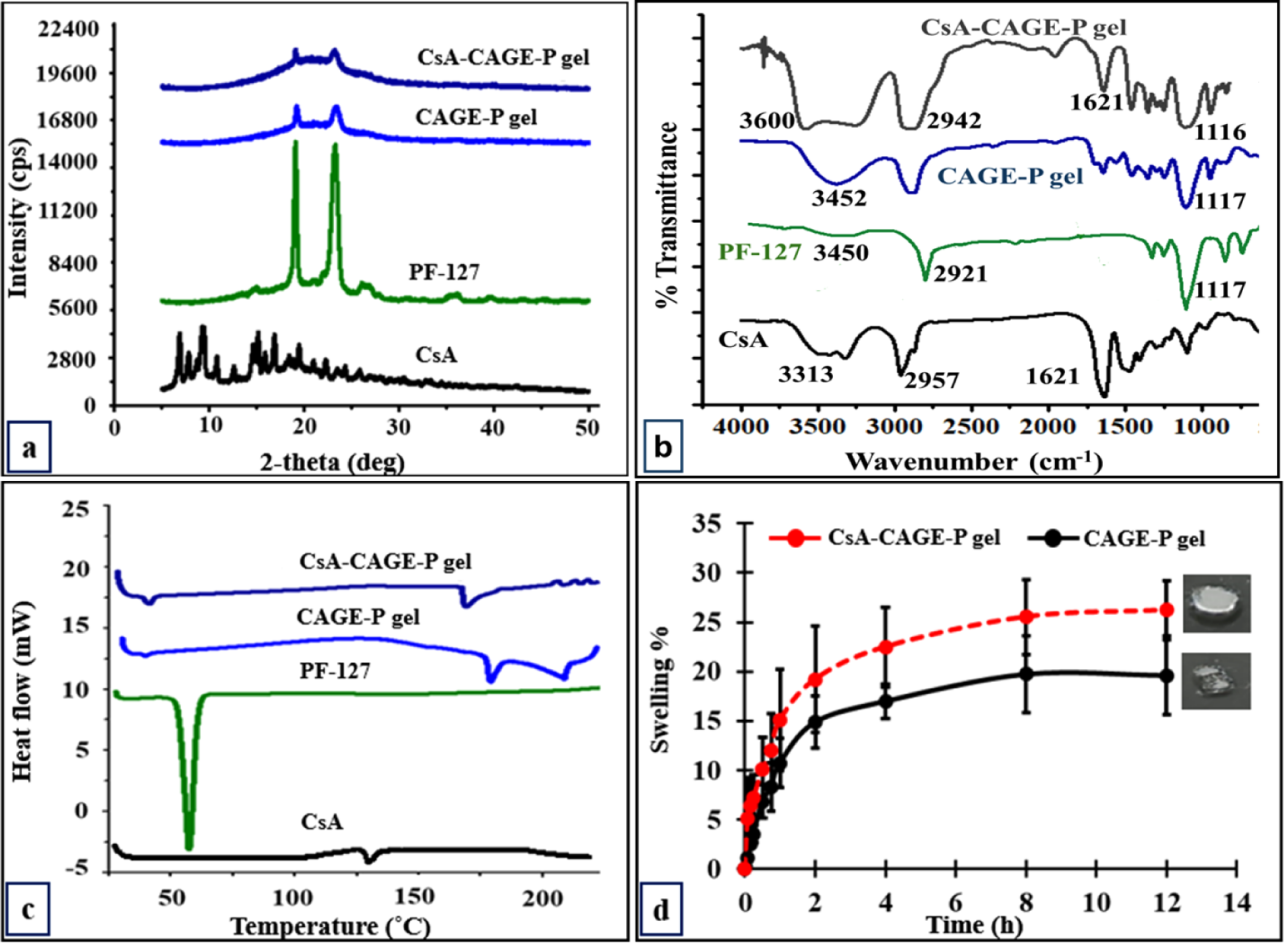
Representative powder X-ray diffractograms showing the amorphous or crystalline nature of (a). FTIR spectra (b) and DSC thermograms (c) showing the characteristic spectral bands and melting temperature for neat CsA, Pluronic F127, CAGE-P gel, and CsA-CAGE-P gel. Swelling behavior of blank CAGE-P gel and CsA-CAGE-P gel in phosphate-buffered saline (pH 7.4, 37 °C). The inset image shows the time-dependent swollen behavior of gel system (d). Data represent the mean ± SD (*n* = 4).

The gel formulation turned from white opaque to yellow after the addition of CAGE and CsA. The CsA-CAGE-P gel was found to be homogeneous with no particulates. The pH values of CsA-CAGE-P and blank CAGE-P gel were found to be 5.2 ± 0.61 and 4.83 ± 0.21, respectively. The X-ray diffraction pattern of neat CsA, Pluronic F127, CAGE-P, and CsA-CAGE-P gel is shown in Figure 1a. The neat CsA was found to be in a crystalline state with diffraction peaks at 2θ values of 6.78°, 9.12°, 15.06°, and 16.80°. The X-ray diffractogram of neat Pluronic F127 showed distinct peaks at 2θ values of 18.89° and 23.09°. CAGE-P and CsA-CAGE-P gel formulations showed peaks with reduced intensity at 2θ values of 18.89° band and 23.09°, similar to Pluronic F127. There were no characteristic diffraction peaks of CsA observed in the CsA-CAGE-P gel. Figure 1b shows representative FTIR spectra of neat CsA, Pluronic F127, CAGE-P gel formulations, and CsA-CAGE-P gel formulations. For CsA, the characteristic bands were shown at 3313 cm^–1^ (N–H stretching), 2957 cm^–1^ (C–H stretching), and 1621 cm^–1^ (C=O stretching) vibrations.^42^ The absorption band intensity in the region 2921 and 1117 cm^–1^ corresponds to the aliphatic C–H stretching vibrations and C–O stretching vibrations, respectively. 3450 cm^–1^ resembles OH-stretching vibrations in Pluronic (PF127).^43^ Blank CAGE-P gel is a mixture of choline geranate and PEG in the PF127 gel formulation. The shift in the broad peak from 3300 to 3452 cm^–1^ can be attributed to the combination of PF127 with the choline–OH bond and carboxylic acid in geranic acid.^44^ Low intensified peaks at 1381 and 1360 cm^–1^ (C–H deformation peak) indicated the existence of a −CH_2_ chain segment of the PEG.^45^ For the CsA-CAGE-P gel, the characteristic peaks of CsA were retained, revealing the CsA loading in the CAGE-P gel matrix. Notably, the most intensified characteristic peak of CsA (1621 cm^–1^) was observed in CsA-CAGE-P gel, which was absent in neat PF127 and blank CAGE-P gel. A slight hypsochromic shift of the characteristic peak of CsA (2957 cm^–1^) to 2942 cm^–1^ can be attributed to structural changes in the polymeric organogel network and could be a consequence of loading of CsA in CAGE-P gel.^46−48^ However, the presence of broader peaks at 3600 cm^–1^ in the CsA-loaded CAGE-P gel confirmed immense intermolecular hydrogen bonding.

If there is a sudden and significant change in the thermal characteristics of either the drug or polymer, it could suggest a potential drug–polymer interaction. Figure S5a shows the endothermic melting peak of CsA and PF127 with a hypsochromic shift and lower intensity at 127 and 56 °C in comparison to the pure drug and polymer peak, suggesting that the crystalline nature of both the components was retained. The findings from the FTIR studies showed that all the characteristic infrared absorption peaks of CsA and PF127 at 1117 cm^–1^, 1621 cm^–1^, and 2921 cm^–1^ were retained in the physical mixture of CsA + PF127, with no significant shifting, which indicated that no interaction occurred between CsA and PF127 (Figure S5b). Therefore, the drug and polymer were compatible to form a stable gel formulation.

Figure S6a,b shows the images captured and percentage porosity for the CsA-loaded CAGE-P and CAGE-P organogel formulations at different time intervals. The percentage porosity was 31.75 ± 1.06% and 33 ± 0.97% for the CsA-CAGE-P gel and the CAGE-P gel, respectively. Figure 1c shows representative DSC thermograms of neat CsA, Pluronic F127, CAGE-P, and CsA-CAGE-P gel. Neat CsA and Pluronic F127 showed endothermic transitions at 132.3 and 57.3 °C, respectively. The endothermic peak at 41.4 and 40 °C for both CAGE-P and CsA-CAGE-P gel is because of the presence of PEG 400 in gel formulation.^49^ The endothermic peaks within 160–200 °C in CAGE-P and CsA-CAGE-P gels are attributed to the interaction of CAGE in the gel base. The disappearance of the characteristic endothermic transition of CsA in CsA-CAGE-P gel formulation indicates its phase change to an amorphous form.^49,50^ The swelling index was determined in PBS at 37 °C to understand the solvent uptake by gel. Figure 1d shows the increase in the swelling index of the CsA-CAGE-P gel with the increase in incubation time from 0 to 12 h. However, after 14 h, the gel matrix started to disintegrate slowly with an increase in the time. The maximum swelling index of CsA-CAGE-P gel and CAGE-P gel in 12 h was 26.2 ± 3% and 19.7 ± 4%, respectively.

**Figure 2:**
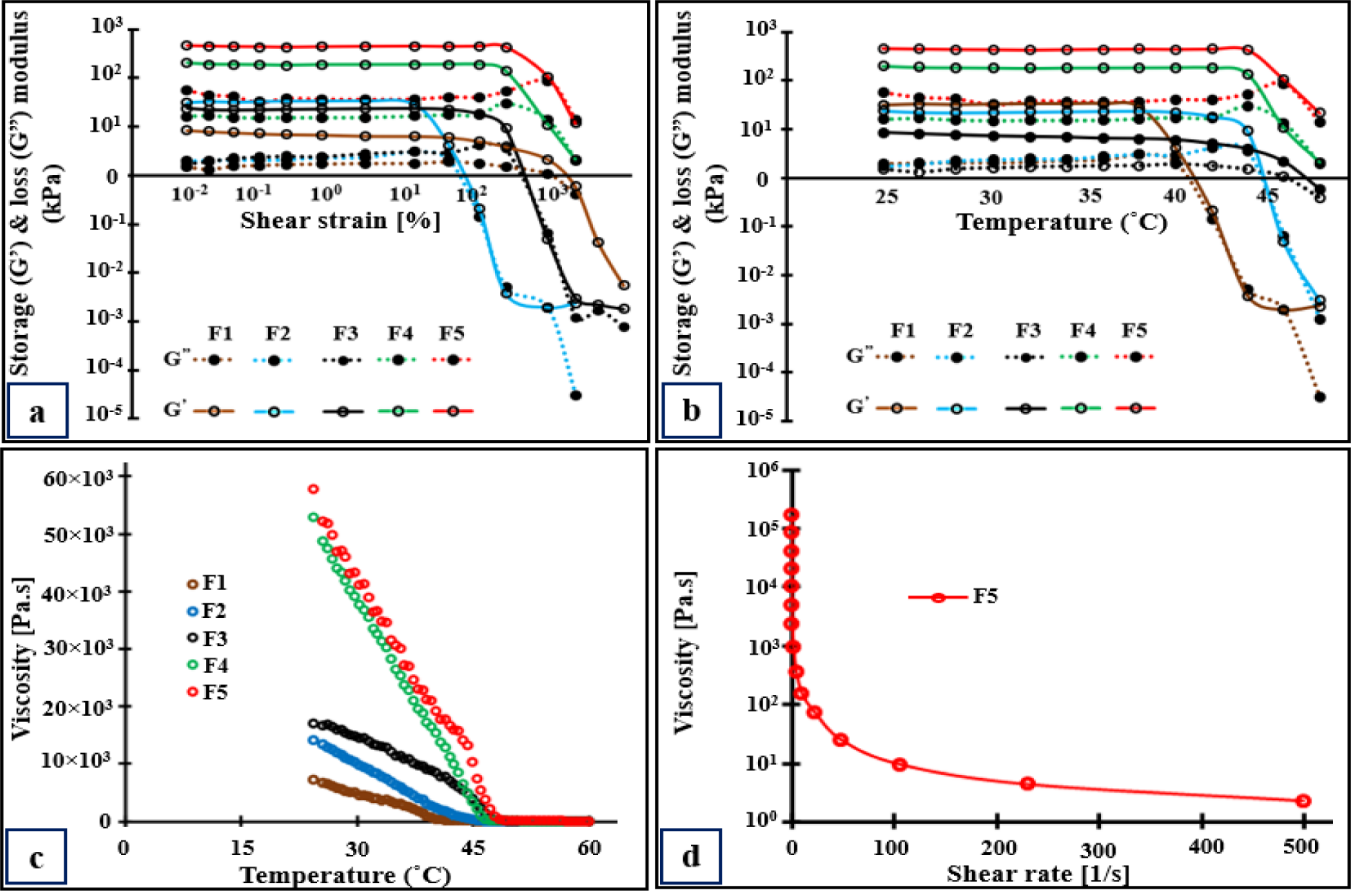
Rheological analysis of formulations. Storage (*G*′) and loss (*G*″) modulus of CsA-CAGE-P gel was determined in amplitude (a) and temperature (b) sweep studies. Change in viscosity with the increase in temperature (c) and varying shear rate (d). Data represent a mean of 3–4 repetitions. Refer to Table 1 for formulation composition.

The rheological parameters including elastic (*G*′) and loss (*G*″) moduli and viscosity (η) were determined for gel formulations. Figure 2a shows the linear viscoelastic region of the different gel formulations. The shear strain was fixed at 1% for the remaining rheological studies. The gel-to-sol transition temperature for Pluronic F188 gel (4.6% w/v) was found to be 39 ± 2.8 °C. The transition temperature increased to 42.5 ± 2.3 °C for Pluronic F127 gel (4.6% w/v). For CsA-CAGE-P gel (F5) containing 22.7% (w/v) Pluronic F127, the transition temperature was found to be 45.8 ± 1.31 °C (Figure 2b). The *G*′ and *G*″ values increased with the increase in Pluronic F127 concentrations from 4.6 to 22.7% w/v (Figure 2b). The storage modulus was greater than the loss modulus indicating the gel phase was below 40 °C. Figure 2c shows a decrease in viscosity with the increase in temperature for all of the formulations indicative of shear thinning properties. Figure 2d shows a decrease in viscosity with the increase in the shear rate for the CsA-CAGE-P gel. Figure S7 shows the increase in shear stress with an increase in the shear rate at 25 °C.

**Figure 3:**
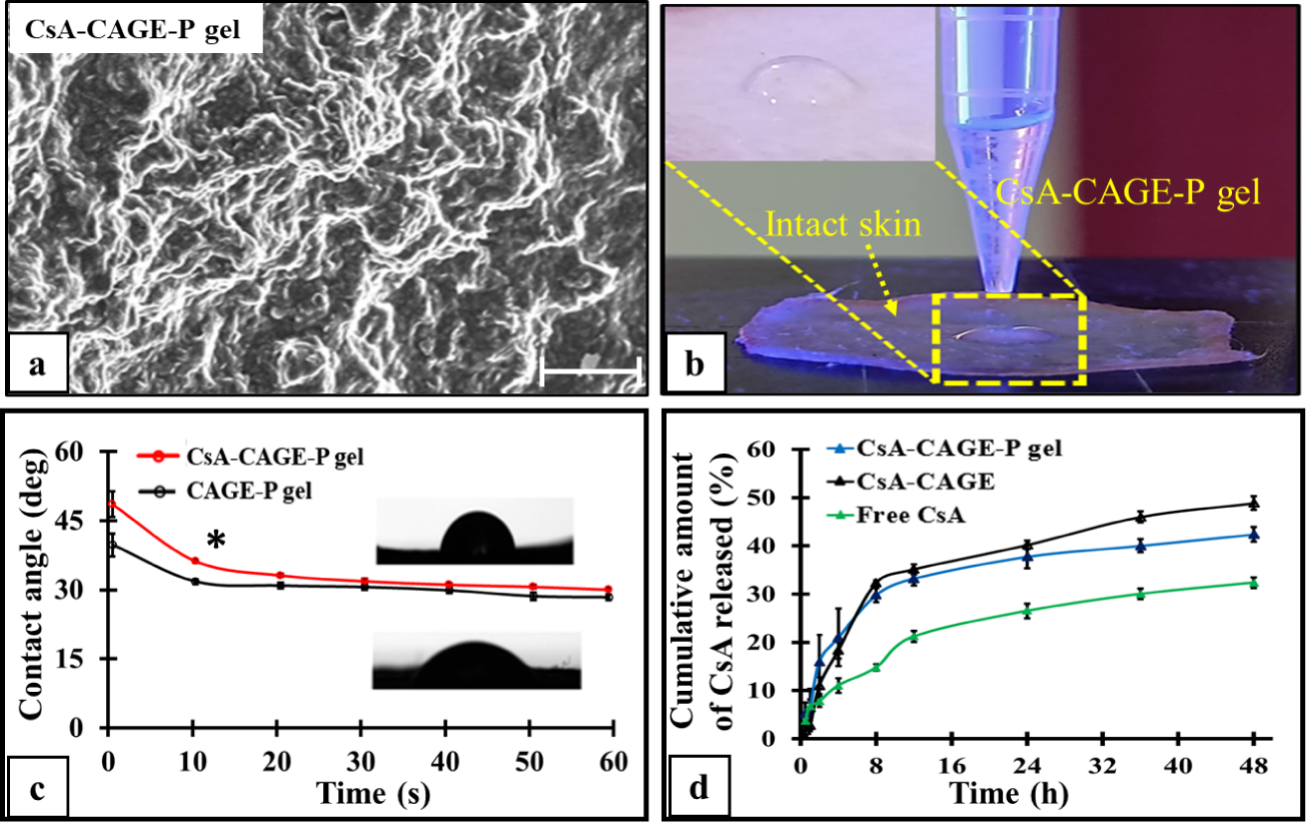
Scanning electron microscopic (SEM) images of CsA-CAGE-P gel formulation, depicting the wrinkled networks (magnification 600×) (a). Experimental setup for the surface wettability and spreading measurement employing the contact angle of gel using a goniometer on the excised intact porcine skin of thickness, 0.45–0.48 mm (b). The porcine skin surface has a different wettability for both CsA-loaded and blank CAGE-P gel formulations with a change in the contact angle with time. The inset images show the sessile droplet formation for the CsA-CAGE-P gel (upper) and blank CAGE-P gel (lower) formulation (c) and *in vitro* release profile of CsA from different formulations at different time intervals (d). Data represent the mean ± SD (*n* = 4). The asterisk (*) represents that the value is significantly different at *p* < 0.05 compared to blank CAGE-P gel formulation. The scale bar represents 50 μm.

Figure 3a shows a scanning electron microscopic image of the CsA-CAGE-P gel. Figure 3b shows the experimental setup for the contact angle measurement. Figure 3c shows a change in the contact angle with the progression of time. The gel formulations showed a contact angle of <50° on the skin sample.

The amount of CsA present in one gram of CsA-CAGE and CsA-CAGE-P gel was found to be 9.71 and 7.73 mg, respectively.

Figure 3d shows the release profile for CsA from the CAGE and CAGE-P gel formulations. The percentage of CsA diffused across the dialysis membrane from CAGE and CAGE-P gel was found to be 48.8 ± 1.4% and 42.4 ± 1.5%, respectively, at the end of 48 h. The percentage of free CsA diffused was 29.8 ± 1.83% in 48 h. The initial release profile of CsA from CsA-CAGE-P gel showed a burst release phase, where 21 ± 5.9% was released in 4 h. The release of CsA from formulations was fitted into kinetic models (Table S2).

### 3.2 *Ex Vivo* Skin Permeation of CsA in Porcine Ear Skin

The excised porcine ear skin used for *ex vivo* permeation studies was measured and showed an average thickness of 380 ± 10.3 μm. The average TEWL and TEER for the intact excised porcine ear skin were found to be 8.1 ± 1.1 g/m^2^/h and 16.2 ± 1.4 kΩ/cm^2^, respectively. The TEWL values of skin samples increased from 8.1 ± 1.1 to 38.6 ± 0.72 g/m^2^/h after 48 h of incubation with CAGE-P gel. Similarly, TEER values decreased from 16.2 ± 1.32 to 3.1 ± 0.6 kΩ/cm^2^ after 48 h of incubation with CAGE-P gel.

HPLC chromatograms of CsA extracted from porcine skin are shown in Figure S3b. The CsA retention time was found to be 3.4 min, compared to 3.2 min for free CsA. The minimum quantitation level was 1 μg/mL, with a percentage CsA recovery of 84–90%.

**Figure 4:**
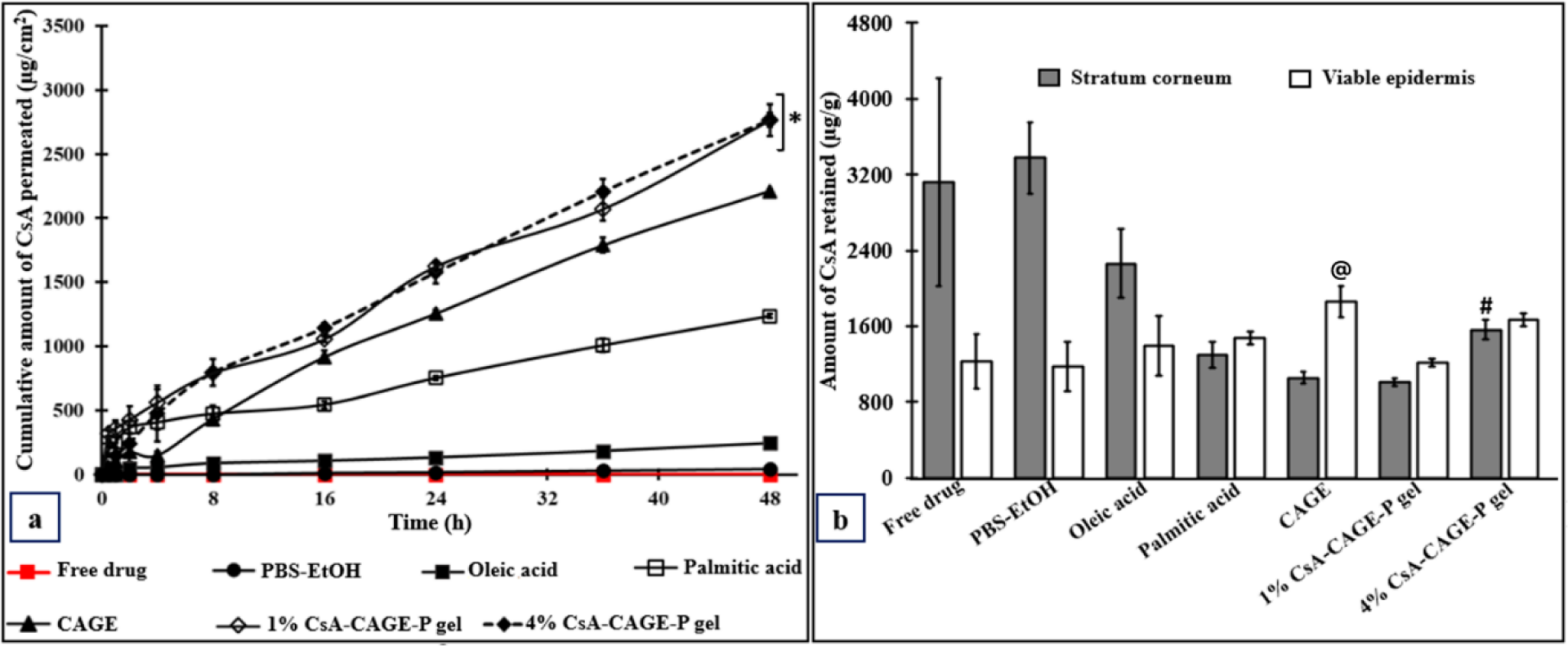
Effect of permeation enhancers on the skin permeation and retention of CsA. Permeation profile of CsA across the skin (a) and its retention within the skin after 48 h of application in different formulations (b). Data represent the mean ± SD (*n* = 4–6). The asterisk (*) represents that the values of 1% or 4% CsA-CAGE-P gel are significantly different at *p* < 0.05 compared to all the other groups. “@” represents that the retention of CsA in viable skin is significantly more at *p* < 0.05 compared to all the other groups. “#” represents that the retention of CsA in SC is significantly more at *p* < 0.05 compared to all the other formulations, except free CsA or PBS-EtoH or OA-treated groups.

According to *ex vivo* skin permeation studies, neat CsA did not permeate across intact porcine skin for up to 48 h (Figure 4a). However, the amount of CsA retained in SC and viable skin were found to be 3121 ± 1093 and 1230 ± 291 μg/g, respectively (Figure 4b). Figure 4a shows permeation profiles of CsA in the excised porcine skin applied with PBS–ethanol (65:35), OA (0.25% v/v), PA (0.25% w/v), and CAGE and CAGE-P gel for 48 h. Table 2 shows the permeation parameters of CsA transport across the skin. After application with a PBS–ethanol combination, the cumulative amount of CsA that permeated across the skin in 48 h (Q48) was found to be 42 ± 1.04 μg/cm^2^. The lag time and the diffusion coefficient were found to be 4.8 ± 0.66 and 0.070 ± 0.009 (×10^–3^ cm^2^/h), respectively. The CsA applied with OA and PA showed significantly greater *Q*_48_ at 244 ± 5.03 μg/cm^2^ and 1236 ± 17.24 μg/cm^2^, respectively, compared with the PBS–ethanol mixture. Application of CsA-CAGE further increased the *Q*_48_ to 2211 ± 14.12 μg/cm^2^. Compared to applications of CsA in OA, PA, and PBS–ethanol; CAGE gave the highest flux, diffusion coefficient, and penetration coefficient (Table 2). Therefore, CAGE was chosen to be incorporated into the Pluronic F127 gel. The incorporation of CAGE in Pluronic F127 gel further enhanced the *Q*_48_, flux, diffusion coefficient, and permeation coefficient of CsA. The increase in CsA concentration from 1% to 4% (saturated condition) in CAGE-P gel showed the greatest permeation through the skin. However, the lag time and diffusion coefficient for the CsA applied with different enhancers (OA, PA, and CAGE) or Pluronic gel were calculated to be 0.

**Table 2:** Skin Permeation Parameters of CsA after Treatment with Different Formulations^a^

Formulation	*J* (μg/cm^2^/h)	*K*_p_ (×10^–3^cm/h)	*Q*_48_ (μg/cm^2^)
Untreated skin	-	-	-
CsA (PBS–ethanol)	0.96 ± 0.05	0.48 ± 0.02	42 ± 1
CsA-OA	4.3 ± 0.2	2 ± 0.1	244 ± 5
CsA-PA	21 ± 2.1	10 ± 1	1236 ± 17
CsA-CAGE	46.3 ± 1.1	23 ± 0.5	2211 ± 14
CsA-CAGE-P gel (1%)	56.9 ± 3	28 ± 1	2766 ± 55*
CsA-CAGE-P gel (4%)	52.1 ± 1.75	26 ± 0.8	2764 ± 125

a ***J***, flux; ***K*_p_**, permeability coefficient; and ***Q*_48_**, the cumulative amount of CsA permeated at the end of 48 h per unit area. The thickness of the intact skin ranged from 0.40 to 0.45 mm. Data represent the mean ± SD (*n* = 4). “*” represents that the values are significantly different at *p* < 0.05 compared to other CsA-treated groups, except 4% CsA-CAGE-P gel.

Figure 4b shows the amount of CsA retained within the SC and viable skin after application in different formulations for 48 h. Application of CsA in CAGE significantly enhanced the amount of CsA retained within the viable skin (1861 ± 165.1 μg/g) compared to OA and PA application. In addition, CsA retained within the SC and viable skin further increased to 1561 ± 101.5 and 1673 ± 67.20 μg/g after application of 4% CsA-CAGE-P gel formulation.

### 3.3 Mechanistic Studies

**Figure 5:**
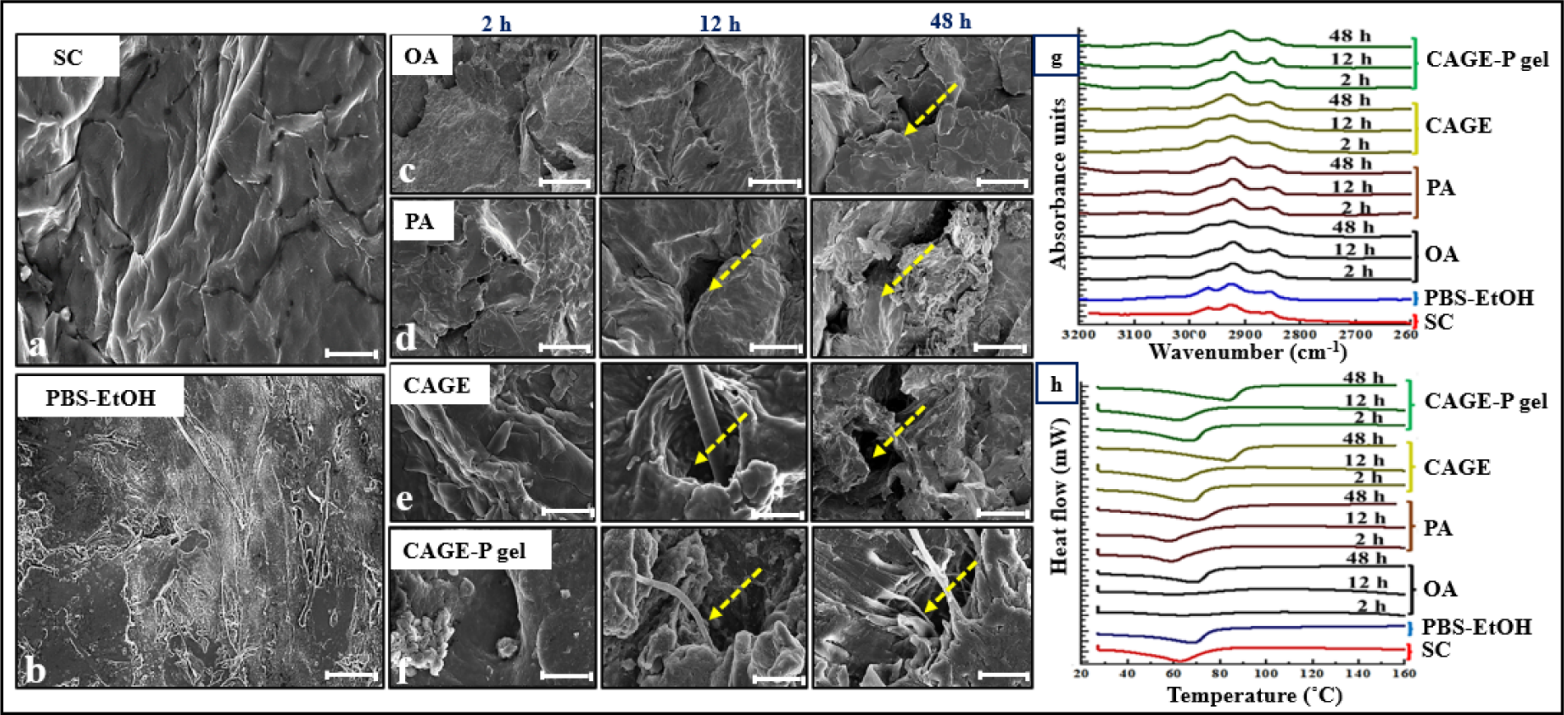
Scanning electron microscopic (SEM) images showing surface morphology for untreated stratum corneum (SC) (a) or treated with PBS–ethanol after 48 h (b). The SEM images captured and arranged from left to right are of SC treated with oleic acid (c); palmitic acid (d); and CAGE (e) and CAGE-P gel (f) at 2, 12, and 48 h, respectively. The arrows indicate cracks and skin furrows. FTIR spectra (g) and DSC thermograms (h) of SC treated with permeation enhancers were obtained at different time points. The scale bar represents 50 μm. **OA**: oleic acid; **PA**: palmitic acid; **CAGE**: choline bicarbonate and geranic acid mixture; **CAGE-P gel**: Pluronic gel.

The TEWL and TEER values prior to and following treatment with PBS–ethanol, OA, PA, CAGE, and CAGE-P gel at different time points are shown in Table 3 for comparison. The TEWL value for the excised porcine skin was less than 10 g/m^2^/h. In comparison to untreated skin, the application of permeation enhancers to the skin increased TEWL values significantly (*p* < 0.05). The TEWL values were highest after 12 and 48 h for the skin samples treated with CAGE-P gel. The TEER values of untreated skin samples were more than 12 kΩ/cm^2^. The TEER values significantly decreased to ≤6 kΩ/cm^2^ at *p* < 0.05, after co-treatment with CAGE or CAGE-P gel at the end of 48 h compared to the untreated skin. Figures 5a–f shows SEM images of the untreated skin sample and the skin samples treated with different chemical permeation enhancers for 2, 12, and 48 h. The untreated skin surface was smooth without fissures and pores (Figure 5a). The treatment with permeation enhancers altered the skin surface to a different degree. The treatment with CAGE and CAGE-P gel created wider fissures and pores on the skin surface (Figure 5e,f).

**Table 3:** Effect of Chemical Permeation Enhancers on the TEWL and TEER of Excised Porcine Skin^a^

	**TEWL** (g/m^2^/h)			**TEER** (kΩ/cm^2^)		
Formulations	Before	After	Percentage (%) increase	Before	After	Percentage (%) decrease
Untreated skin-48 h	8.1 ± 1.1	18.5 ± 0.65	128 ± 4	16.2 ± 1.4	7.4 ± 1.4	54 ± 10
Control (PBS + ethanol)-48 h	7.8 ± 0.3	23.0 ± 0.1	195 ± 1	13.0 ± 0.8	6.01 ± 0.1	53 ± 1
OA-2h	8.1 ± 1.1	20.0 ± 1.7	147 ± 12	14.2 ± 1.9	8.11 ± 1.3	42 ± 7
OA-12h	7.5 ± 1.1	28.3 ± 2.2	277 ± 21	15.8 ± 1.5	7.13 ± 1.2	54 ± 9
OA-48h	7.1 ± 0.7	32.5 ± 1.1	358 ± 12	14.8 ± 0.9	6.10 ± 1.9	59 ± 18
PA-2h	7.3 ± 1.4	21.8 ± 1.7	199 ± 15	14.5 ± 1.2	7.10 ± 0.4	51 ± 3
PA-12h	7.8 ± 1.3	30.2 ± 0.5	287 ± 4	14.1 ± 1.8	6.71 ± 0.5	52 ± 4
PA-48h	8.5 ± 0.4	34.5 ± 0.7	306 ± 6	13.5 ± 0.4	6.50 ± 1.4	52 ± 11
CAGE-2h	8.4 ± 1.4	26.7 ± 1.3	218 ± 10	14.6 ± 1.6	6.22 ± 1.7	57 ± 15
CAGE-12h	7.8 ± 1.3	32.8 ± 1.1	320 ± 10	14.2 ± 1.5	5.80 ± 1.5	59 ± 15
CAGE-48h	7.2 ± 0.4	36.8 ± 0.3	411 ± 3	14.0 ± 0.2	4.10 ± 1.2	71 ± 21
CAGE-P gel-2h	9.2 ± 1.3	28.8 ± 1.8	213 ± 13	14.8 ± 2.3	5.12 ± 1.5	65 ± 19
CAGE-P gel-12h	8.4 ± 1.7	34.1 ± 1.1	306 ± 9	15.0 ± 0.5	4.11 ± 1.1	73 ± 19
CAGE-P gel-48h	8.2 ± 1.1	38.6 ± 0.7*	371 ± 6	15.3 ± 1.3	3.10 ± 0.6*	80 ± 15

a **TEWL**: transepidermal water loss and **TEER**: transepidermal electrical resistance. Values represent the mean ± SD (*n* = 4–6). Untreated skin was spiked with free cyclosporine A, without any enhancers. “*” represents that the TEWL and TEER values are significantly different at *p* < 0.05 compared to other treated groups after 48 h.

Figure 5g displays the FTIR spectra of SC before and after treatment with enhancers at different time intervals. The asymmetric (2920 cm^–1^) and symmetric (2850 cm^–1^) C–H stretchings associated with lipids within the SC were recorded. The shift to higher wavenumbers was observed after treatment with the enhancers. The treatment of SC with PBS–ethanol showed a negligible shift in peak positions compared with untreated SC. The SC samples incubated in CAGE and CAGE-P gel showed reduced peak heights at 2920 and 2850 cm^–1^ after 12 and 48 h indicating lipid extraction.^51,52^

Figure 5h and Table 4 show the results of thermal analysis of skin samples after treatment with PBS–ethanol, OA, PA, CAGE, and CAGE-P gel at different time points. The transition temperature for the untreated skin sample was found to be 70.8 ± 0.81 °C, and the change in enthalpy (*H*) was found to be −641.7 ± 4.71 J/g. The transition temperature of skin samples increased to 81.2 ± 0.43, 77.3 ± 0.75, 65.4 ± 1.70, and 82.1 ± 2.11 °C after 48 h incubation with OA, PA, CAGE, and CAGE-P gel, respectively. The incubation of skin with PBS–ethanol (65:35) decreased the enthalpy change to −521.7 ± 24.71 J/g and increased the transition temperature to 73.7 ± 0.58 °C. In general, Δ*H* decreased after treatment with OA, PA, CAGE, and CAGE-P gel. The Δ*H* for thermal transition of skin decreased significantly (*p* < 0.05) after incubation with CAGE and CAGE-P gel at 12 and 48 h compared to untreated skin.

**Table 4:** Thermal Analysis of the Stratum Corneum before and after Treatment with Chemical Permeation Enhancers^a^

Formulations	Transition temperature (°C)	Enthalpy change (−Δ*H*, J/g)
Stratum corneum (SC)	70.8 ± 0.8	641.7 ± 4.7
Control (PBS + ethanol)-48 h	73.7 ± 0.5	521.7 ± 24.7
OA-2h	68.6 ± 0.5	421.7 ± 7.5
OA-12h	66.6 ± 1.1	280.4 ± 5.5
OA-48h	81.2 ± 0.4	142.7 ± 10.1
PA-2h	61.9 ± 1.6	264.5 ± 4.1
PA-12h	60.4 ± 0.7	54.33 ± 4.1
PA-48h	77.3 ± 0.7	48.70 ± 2.5
CAGE-2h	61.4 ± 1.4	216.6 ± 5.9
CAGE-12h	57.3 ± 1.1	59.80 ± 1.5
CAGE-48h	65.4 ± 1.7	41.20 ± 1.6
CAGE-P gel-2h	65.1 ± 2.1	200.2 ± 5.8
CAGE-P gel-12h	59.3 ± 1.8	49.10 ± 3.5
CAGE-P gel-48h	82.1 ± 2.1	37.70 ± 2.8*

a Values represent the mean ± SD (*n* = 3). “*” represents that the value is significantly different compared to other groups at *p* < 0.05.

### 3.4 Pharmacokinetic Analysis

The plasma concentrations of CsA after topical application in different formulations were determined by the developed RP-HPLC method. The lowest limit of quantitation for CsA was found to be 0.37 ng/mL. The extraction of CsA from plasma was optimized from different concentrations ranging from 0.00037 to 18.75 μg/mL. The average percentage of recovery of CsA from rat skin was found to be 87.2%.

**Figure 6:**
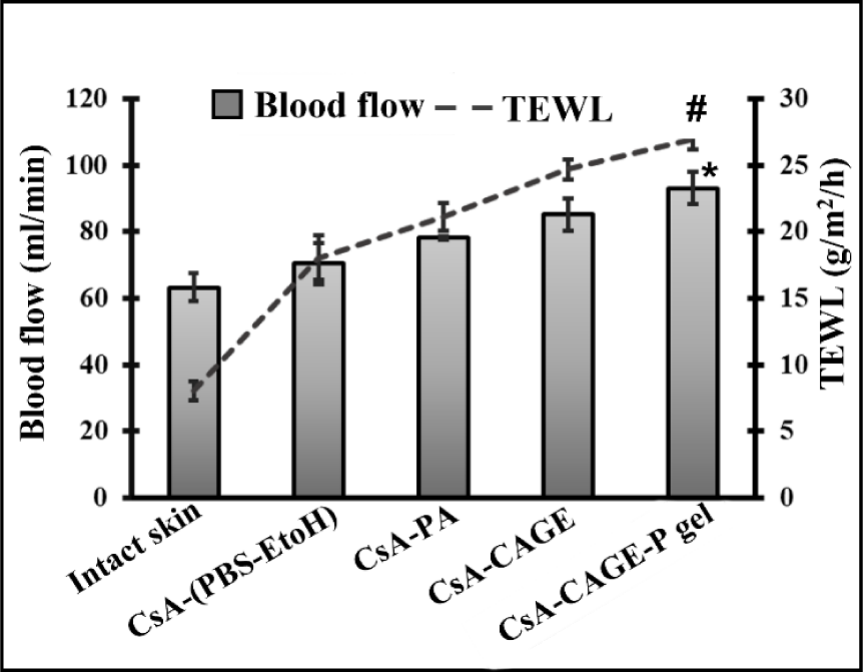
TEWL and LDF values for intact and treated skin were measured after 2 h of application of different formulations. Values represent the mean ± SD (*n* = 4). “*” and “#” represent that the value is significantly different at *p* < 0.05 compared to all the other groups.

Before and after application of CsA in different formulations to the rat dorsal skin, TEWL and laser Doppler blood flow rates were assessed. Figure 6 shows TEWL values of the rat skin after 2 h of application of CsA in different formulations. All the formulations caused significant (*p* < 0.05) increases in TEWL values compared with untreated skin. However, application of CsA in CAGE-P gel for 2 h increased TEWL by 3.3-fold compared with the untreated skin.

The laser Doppler flowmeter allows continuous recording of blood flow within the skin, which would be indicative of skin irritation after the application of chemical enhancers. The normal blood flow rate in rats was found to be 63.21 ± 4.17 mL/min. The blood flow rate increased after 2 h application of PBS–ethanol mixture and PA compared with the untreated skin (Figure 6). However, the blood flow rate increased significantly (*p* < 0.05) to 85.2 ± 4.83 and 93.2 ± 4.96 mL/min after 2 h application of CsA-CAGE and CsA-CAGE-P gel, respectively, compared with the untreated skin.

**Figure 7:**
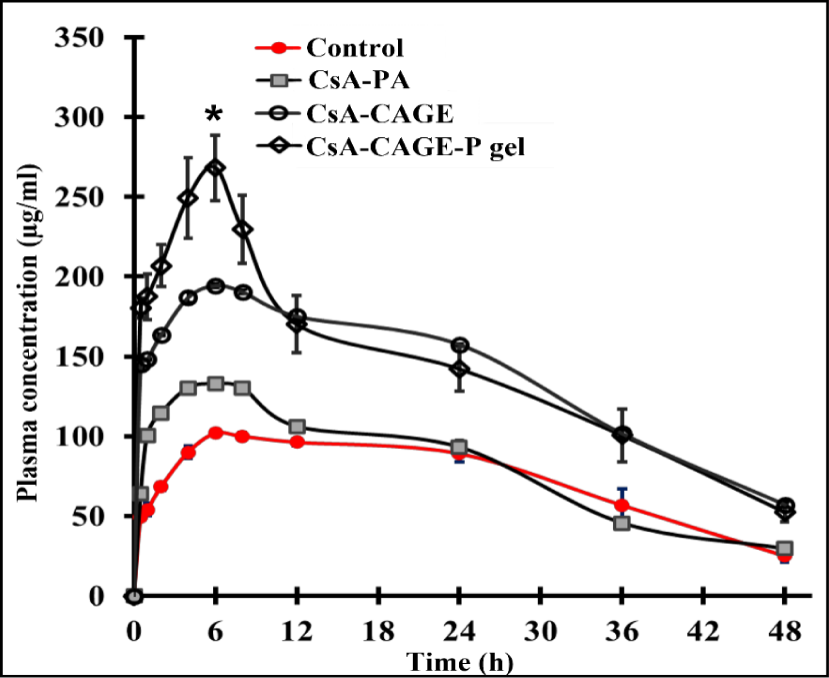
Plasma concentration after topical application of CsA in different formulations. Values represent the mean ± SD (*n* = 4). The asterisk (*) represents that the value is significantly different at *p* < 0.05 compared to all the other groups.

The mean plasma concentration of CsA after topical application in a rat model is depicted as a time profile in Figure 7. The pharmacokinetic parameters for CsA delivered in different formulations are shown in Table 5 and were calculated by using WinNonlin software (Pheonix, Certara, USA). The control group applied to CsA dissolved in a PBS–ethanol mixture showed a maximum plasma concentration (*C*_max_) of 33.9 ± 1.87 μg/mL/cm^2^ after 6 h of topical application. The *C*_max_ values were significantly (*p* < 0.05) greater at 132.8 ± 0.312, 194.3 ± 1.137, and 269.2 ± 18.33 μg/mL with the application of CsA in PA, CAGE, and CAGE-P gel, respectively, compared with the control group. The *t*_max_ remained the same for all of the formulations at 6 h. The AUC_0–48_ after application of CsA-CAGE-P gel was 1.94-fold greater than that of the control group (Table 5).

**Table 5:** Pharmacokinetic Parameters for CsA Topical Application^a^

Parameters	CsA (PBS–ethanol)	CsA-PA	CsA-CAGE	CsA-CAGE-P gel
*K*_e_ (1/h)	0.015 ± 0.003	0.019 ± 0.0	0.024 ± 0.0	0.027 ± 0.003^#^
*t*_1/2_ (h)	13.1 ± 1.12	14.63 ± 0.89	16.45 ± 0.4	18.51 ± 2.41*
*t*_max_ (h)	6	6	6	6
*C*_max_ (μg/mL)	102.8 ± 0.70	132.9 ± 0.13	194.4 ± 1.12	269.2 ± 18.32*
AUC_0–48_ (μg.h/mL)	3523 ± 207.9	3892 ± 24.52	6618 ± 667.8	6821 ± 355.4^#^
AUC_0–inf_ (μg.h/mL)	4002 ± 302.2	4521 ± 26.20	7979 ± 131.0	8231 ± 646.5^#^
*V*_d_ (l/kg)	0.475 ± 0.011	0.326 ± 0.002	0.211 ± 0.0001	0.166 ± 0.007
MRT (h)	25.78 ± 1.5	25.46 ± 0.65	28.73 ± 0.49	28.14 ± 2.37
Clearance (l/h/kg)	0.0073 ± 0.0011	0.0081 ± 0	0.0041 ± 0	0.0045 ± 0.0004

a Data represent the mean ± SD (*n* = 4). “#” represents that the value is significantly different at *p* < 0.05 compared to CsA (PBS–ethanol) and CsA-PA groups. “*” represents that the value is significantly different compared to all the groups at *p* < 0.05.

### 3.5 *In Vitro*–*In Vivo* Correlation

**Figure 8:**
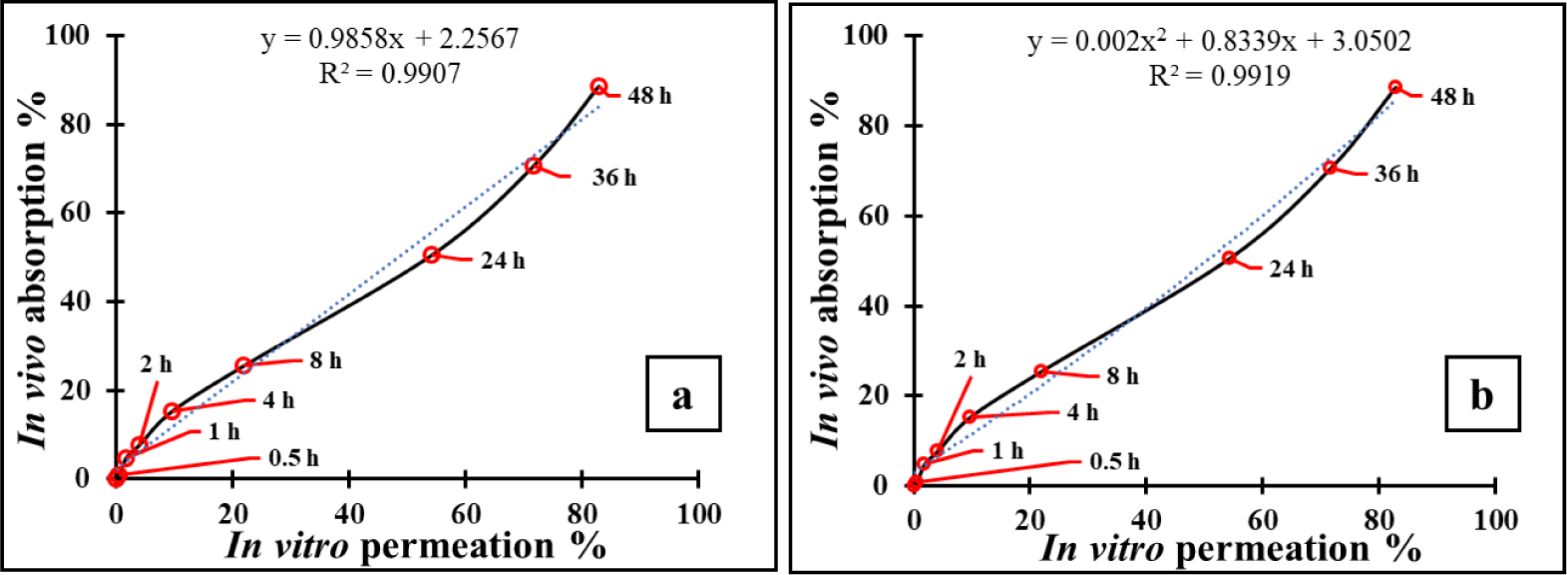
IVIVC model graphing at respective time points showing the linear (a) and polynomial (b) regression, indicating strong *in vitro*–*in vivo* correlation for CsA-CAGE-P gel formulation.

By utilizing the *in vitro* permeability and *in vivo* absorption data obtained from the permeation and pharmacokinetic data for CsA-CAGE-P gel, IVIVC models were developed. A perfect positive correlation was seen between the percentage of *in vitro* permeation (*x* axis) and the percentage of *in vivo* absorption (*y* axis). This relationship is illustrated in Figure 8a where the correlation coefficient (*R*^2^) was found to be 0.991. Furthermore, the polynomial connection seen (*R*^2^ = 0.992) between the *in vitro* and *in vivo* data suggested that the *in vitro* permeation tests can accurately represent physiological conditions that are comparable to those found *in vivo* (Figure 8b).

### 3.6 Efficacy and Histopathological Studies

**Figure 9:**
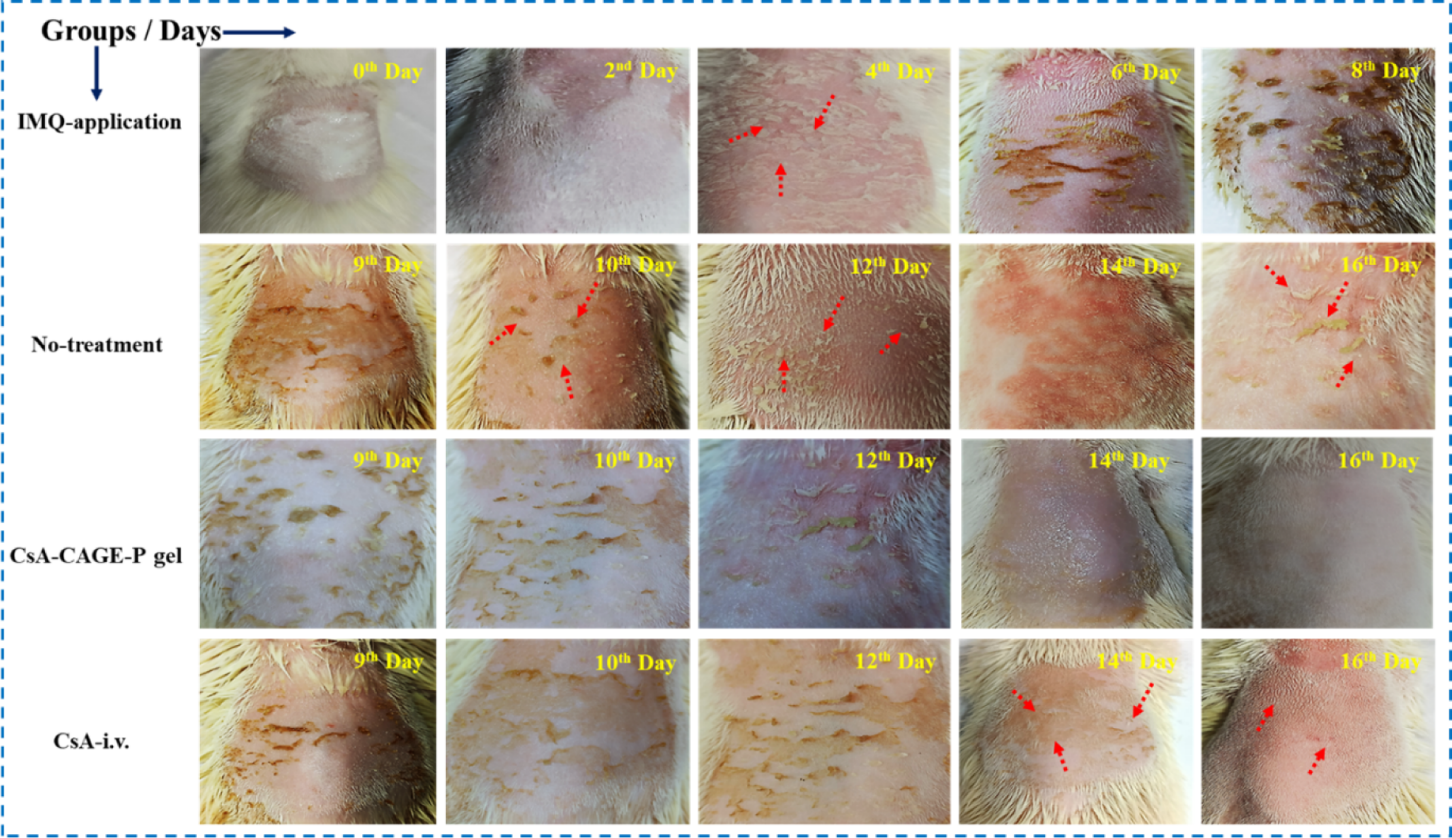
Representative digital images were captured to show the action of topically applied and i.v. administered CsA-CAGE-p gel and CsA solution, respectively, on the macroscopic appearance of the dorsal skin of the IMQ-induced psoriasis rat model at different time intervals for the groups: IMQ-application (2–8 days); no-treatment (9–16 days); CsA-CAGE-P gel (9–16 days); and CsA-i.v. (9–16 days). The red arrow indicates the appearance of a scaly buildup of dead skin cells.

The plaque psoriasis model of the rat was developed by applying IMQ to the dorsal skin daily for 8 days. Then, psoriasis was treated by applying an optimized CsA formulation, a CsA-CAGE-P gel. The performance of the topical CsA-CAGE-P gel was compared to intravenously administered CsA. The intravenous dose of CsA was prepared by dissolving 1% CsA in polyoxyethylated castor oil (65% w/v) and ethanol (35% w/v). Figure 9 shows the digital images of rats applied with IMQ to induce plaque psoriasis. IMQ-induced psoriasis was manifested by scaly lesions, erythema, and increased skin thickness. The skin surface of the IMQ-applied group appeared darker. CsA-CAGE-P gel application resulted in a complete reduction in plaques after 8 days. In contrast, intravenous administration of CsA did not show complete reversal of plaque formation.

**Figure 10:**
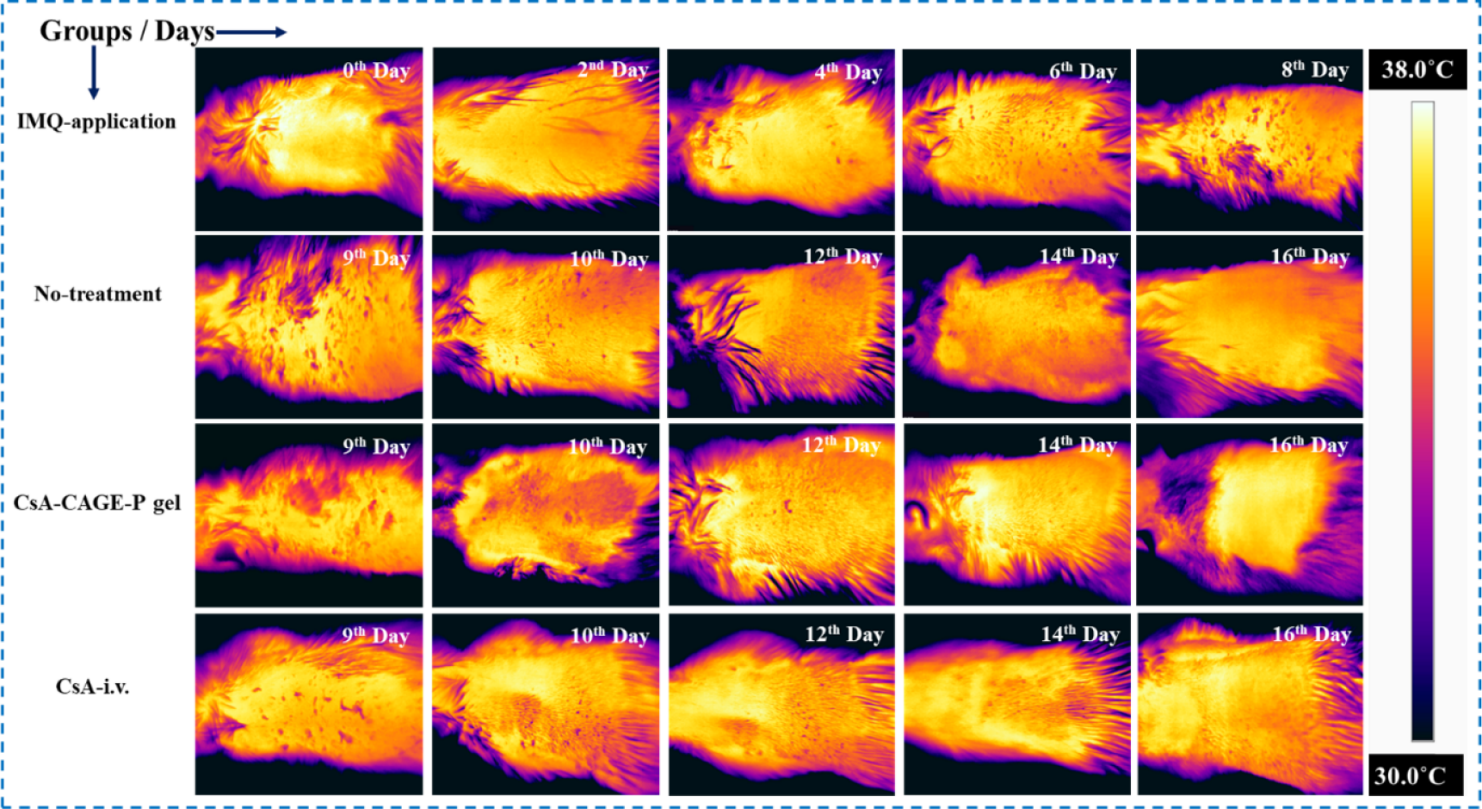
Representative infrared thermal images were captured to show the action of topically applied and i.v. administered CsA-CAGE-P gel and CsA solution, respectively, on the dorsal skin of the IMQ-induced psoriasis rat model. The row indicates the temperature of the skin tissue at different time intervals for the groups: IMQ-application (2–8 days); no-treatment (9–16 days); CsA-CAGE-P gel (9–16 days); and CsA-i.v. (9–16 days).

Figure 10 shows the infrared thermal images of the psoriatic, untreated, and treated skin. The normal skin temperature measured in the control group was found to be 37.4 ± 0.36 °C. IMQ application for 8 days significantly (*p* < 0.05) decreased the skin temperature to 33.2 ± 0.63 °C (Figure S8). The application of CsA-CAGE-P gel and intravenous CsA administration increased the body temperature to 36.3 ± 0.84 °C and 34.1 ± 1.35 °C at the end of the 16th day. However, the body temperature for the untreated group remained at 33.6 ± 1.42 °C even after the 16th day.

**Figure 11:**
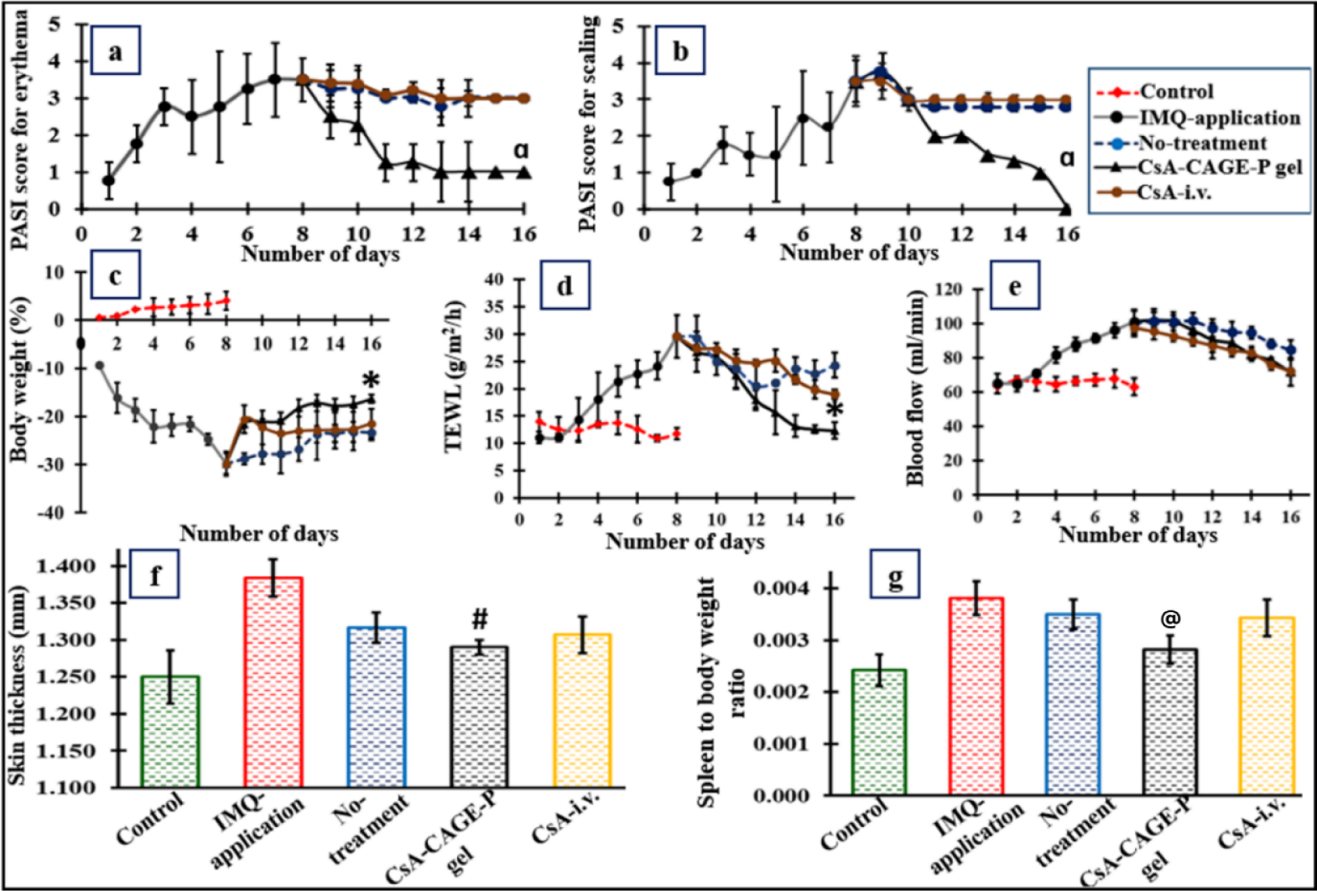
PASI scores of the plaque psoriasis rat model. IMQ cream was applied on the shaved dorsal skin for 8 consecutive days. Rats were either untreated or treated by topical application of CsA-CAGE-P gel or intravenous CsA (CsA i.v.) formulation for 8–16 days. The PASI score for erythema is (a) and scaling (b). Erythema and scaling were scored on a scale from 0 to 4, indicating 0, none; 1, slight; 3, marked; 4, very marked. Change in body weight (c), TEWL (d), and blood flow (e) from 1 to 16 days of psoriasis inducement and treatment. The skinfold on the back of the rat was measured to quantify the thickness of the skin on the 16th day of treatment (f). The effect of CsA-CAGE-P gel or i.v. formulation on IMQ-induced splenomegaly was measured by the spleen-to-body weight ratio (spleen index) (g). Values represent the mean ± SD (*n* = 4). Alpha (α) represents that the value is significantly different at *p* < 0.05 compared to all the other groups. Asterisk (*) represents that the value is significantly different at *p* < 0.05 compared to no-treatment and CsA-i.v. groups. “#” represents that the value is significantly different at *p* < 0.05 compared to IMQ-application or no-treatment groups. “@” represents that the value is significantly different at *p* < 0.05 compared to all the other groups, except the control. Control: without IMQ application (normal skin).

The PASI score is an important parameter to understand the severity of psoriasis. Figure 11 shows the change in the PASI score for erythema and scaling during psoriasis inducement and treatment with CsA formulations. The PASI score increased with the application of IMQ. After treatment with 4% CsA-CAGE-P gel, the PASI scores decreased (Figure 11a,b). Interestingly, the PASI scores did not decrease after treatment with intravenous CsA. The PASI score for erythema and scaling without any treatment was similar to that of the IMQ-applied group. Figure 11c shows the change in body weight of rats during psoriasis inducement and then treatment with CsA formulations. The body weight significantly decreased with IMQ application compared to healthy rats. The greatest recovery in body weight was recorded for the CsA-CAGE-P gel-treated group. Figure S9a,b shows a reduction in water and food intake during IMQ application from the 4th to 8th day. The water and food intake gradually increased from day 9 to 16.

The TEWL and blood flow rate values were found to be 11.7 ± 1.02 g/m^2^/h and 63.14 ± 0.061 mL/min for the control group (Figure 11d,e). The IMQ-applied group showed significantly (*p* < 0.05) greater values of TEWL (29.5 ± 2.01 g/m^2^/h) and LDF (101 ± 7.03 mL/min) after 8 days, attributed to the loss of barrier integrity. The TEWL and blood flow rate values decreased gradually with the application of the CsA-CAGE-P gel.

The skin thickness measured for the control group (healthy skin) was 1.25 ± 0.03 mm. Figure 11f shows that the skin thickness increased to 1.38 ± 0.02 mm after 8 days of IMQ application. The skin thickness was found to be 1.31 ± 0.02, 1.29 ± 0.01, and 1.31 ± 0.02 mm after 16 days for the untreated group and the groups treated with CsA-CAGE-P gel and intravenous CsA, respectively.

Figure 11g shows the spleen-to-body weight ratio. An increase in the spleen weight may reflect an increase in the immune cells in the spleen. To this end, the IMQ-applied group showed the greatest spleen-to-body weight ratio of 0.004 ± 0.0003 compared with all the other groups. Treatment with CsA-CAGE-P gel significantly (*p* < 0.05) decreased the spleen-to-body weight ratio compared with IMQ application. Figure S9c shows digital images of the spleen, indicating the size. The length of the spleen was measured to be 3.8 ± 0.2 cm for IMQ-applied psoriatic rats which was significantly (*p* < 0.05) greater compared to the control group (3.3 ± 0.1 cm). The spleen length decreased to 3.3 ± 0.1 cm and 3.61 ± 0.05 cm after treatment with CsA-CAGE-P gel and intravenous CsA formulations, respectively.

**Figure 12:**
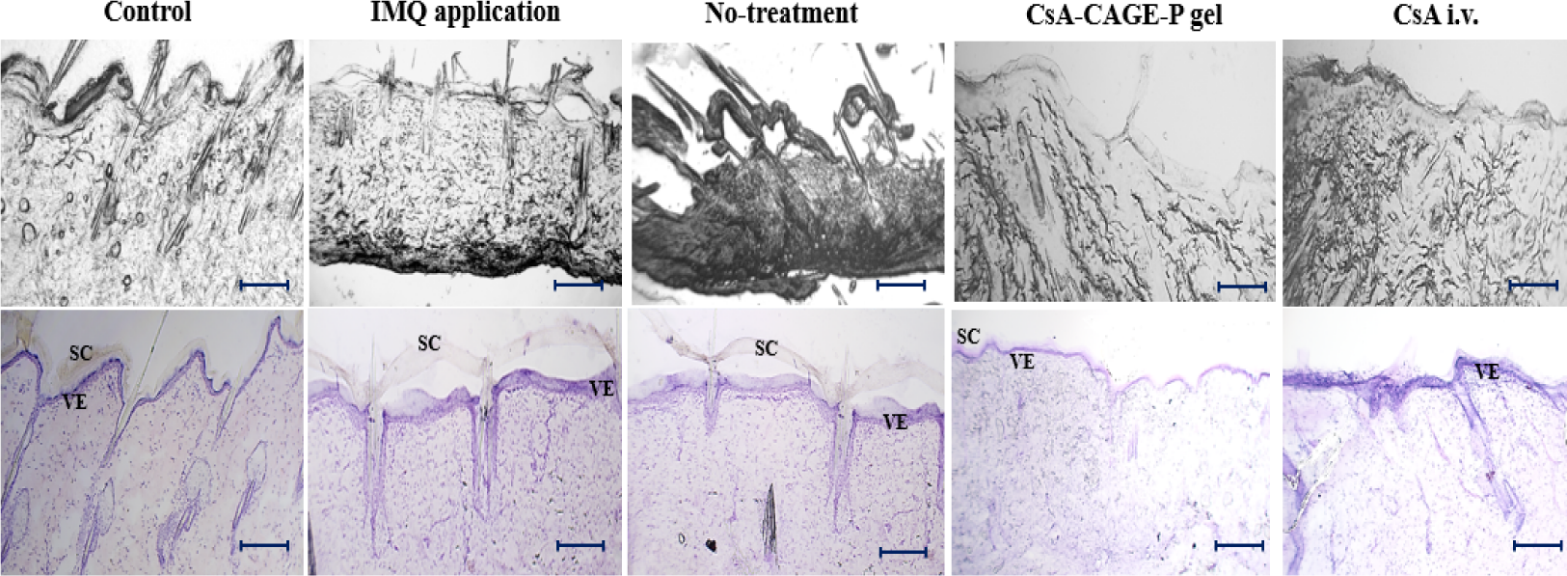
Representative bright-field and hematoxylin and eosin-stained images for the excised skin samples treated with or without different formulations. The IMQ-applied group shows the removal of SC and increased thickness of the epidermis (epidermal hyperplasia) compared to the control group. The same observations were made for the no-treatment and CsA-i.v. administered group without any significant reversal to normal skin conditions. The CsA-CAGE-P gel formulation group shows significant changes to SC and reduced VE thickness. **SC**: stratum corneum; **VE**: viable epidermis. The scale bar is 100 μm.

Figure 12 shows the microscopic images of cryosectioned psoriatic rat skin before and after treatment with different CsA formulations. The control group represents healthy rat skin characterized by the intact SC and epidermal layer (40.5 ± 8.92 μm thickness). The IMQ-applied group showed a thickened epidermis (hyperkeratosis). The untreated rat group did not show a decrease in the epidermal thickness. On the other hand, epidermal thickness reduced to that of normal skin after treatment with CsA-CAGE-P gel.

## 4 Discussion

According to Fick’s first law of diffusion, the steady-state flux of active molecules through the skin is directly related to diffusivity, partition coefficient, and soluble concentration and inversely related to diffusional path length.^53^ Macromolecules such as CsA show poor diffusivity through the skin membrane. Chemical permeation enhancers can be broadly categorized based on their mechanism of action—compounds that improve the thermodynamic activity of active molecules by enhancing saturation solubility within the vehicle and compounds that disrupt the skin barrier to enhance the diffusivity of active molecules.^54^ Furthermore, permeation enhancers can improve the partitioning of active molecules within the skin barrier, SC. The conventional fatty acid-based chemical enhancers act on the SC. OA and PA have been found to fluidize organized lipidic structures within the SC creating transient pores and enhancing the diffusivity of active molecules.^55^ Fatty acids have not been reported to enhance the thermodynamic activity of active molecules.^56,57^ On the other hand, ILs have been found to enhance the saturation solubility of active molecules in addition to altering the SC barrier function.^58^ ILs are deep eutectic systems that are liquids at room temperature. ILs can reduce the melting temperature of solutes.^58^ In the present study, the melting temperature of CsA was reduced from 130.3 to 119.4 °C in the presence of CAGE in a 1:2 ratio (Figure S10). The saturation solubility of CsA was 1.5-fold greater in the presence of CAGE compared to the PBS–ethanol mixture. CAGE showed a similar solubility enhancement for nobiletin and sorafenib in earlier studies.^59,60^ It was found that CAGE forms non-covalent interactions with the active molecules to enhance solubility.^21,51,59,61^

OA and PA disrupt the ordered lipid bilayer interspersed between the corneocytes of SC.^62−64^ Fatty acids embed into the lipids, induce phase change, and enhance fluidity.^65^ The results from thermal analysis, FTIR spectral studies, changes in TEWL and TEER values, and SEM images confirmed alteration of the skin barrier function after incubation with OA and PA. Among PA and OA, PA showed a greater alteration in the barrier function that resulted in greater permeation enhancement of CsA through the skin. The fatty acid, monoolein, showed 2 ± 0.5 μg/cm^2^ cumulative amount of CsA permeated through the porcine skin in 12 h compared with 712 ± 6.09 μg/cm^2^ and 131 ± 5.31 μg/cm^2^ achieved in the presence of PA and OA in the present study.^8^ The addition of CAGE has further altered the barrier nature of the skin compared with PA and OA. The cationic choline and anionic geranic acid penetrate the SC and deliver small molecules and macromolecules including siRNA, bovine serum albumin, ovalbumin, and insulin into the skin.^27,66^ This is evident from the biophysical characterizations. FTIR studies showed lipid extraction after incubation with CAGE. Therefore, CAGE with its barrier disruption property and enhanced saturation solubility provided the greatest flux of CsA through the skin.

The conventional topical formulations are semisolid preparations with greater consistency to allow ease of application and spreadability on the skin.^67^ The semisolid preparations include creams, lotions, ointments, and gels. Gel preparations can be differentiated into hydrogels and organogels. Hydrogels are made of a swollen matrix of a hydrophilic polymer in the presence of water and cross-linker.^68^ In contrast, organogels are formed without the presence of aqueous phase.^14^ CsA being a poorly water-soluble compound would phase separate upon addition to a hydrogel base and would not be the right choice for a topical preparation.^36^ CAGE has been reported to have a viscosity of 568 ± 19 mPa.s.^44^ However, the viscosity was increased to 2235 mPa.s at 500 s^–1^ shear rate and 25 °C for the CsA organogel made of Pluronic F127 and PEG 400. The organogel showed a shear-thinning property. *In vitro* release studies showed a linear relationship, with the highest *R*^2^ value given by the Korsmeyer–Peppas model, followed by Higuchi, zero-order, and first-order models. This model illustrated CsA release from the polymeric Pluronic organogel system, considering a non-Fickian mechanism. Similar were the observations by Musa et al. for the release kinetics of CsA from the nanocolloidal carrier that was developed as a topical treatment for psoriasis.^37^ In general, hydrophobic drugs exhibit low burst release and have a direct correlation between drug log P and the initial burst, where burst release decreases as log P increases.^69^ However, in our studies, the solubility of hydrophobic CsA was significantly increased in the mixture of CAGE and PEG which conversely affected the partition coefficient. We hypothesize that as the hydrophilicity of CsA was increased, its affinity for the hydrophilic polymeric Pluronic gel was also increased, resulting in the burst release of CsA (21 ± 5.9%) from the CAGE-P gel within 4 h.

The final formulation of CsA-CAGE-P gel was found to be clear and stable without phase separation. The saturated concentration of 4% CsA in CAGE-P gel provided the greatest flux and a cumulative amount permeated across the skin. Moreover, the amount of CsA retained within the SC and viable skin were greater for CsA applied using a CAGE-P gel. The present study showed the greatest flux and cumulative amount of CsA permeated across the skin achieved with CAGE-P gel compared with earlier studies that used different permeation enhancers and nanoparticle delivery systems.^4,70^ It is interesting to note that CsA applied in organogel formulation made of CAGE, Pluronic F127 and PEG achieved greater permeation even when compared with neat CAGE. This can be attributed to the activity of CAGE on CsA and skin membranes with and without Pluronic F127 gel. The amount of choline bicarbonate and geranic acid (CAGE) present within the CsA-CAGE-P gel was 9.4% and 21.5% v/v, respectively. The partitioning of permeation enhancers into the skin membrane is efficient when diluted in a suitable vehicle compared with undiluted neat form.^53,71^

Based on the results from *ex vivo* permeation studies, PA, CAGE and CAGE-P gel were selected as permeation enhancers for *in vivo* pharmacokinetic studies. The pharmacokinetic studies in the rat model demonstrated that topical application of CsA-CAGE-P gel had the greatest bioavailability, *C*_max_ and maximum retention time. For comparison, earlier reports showed low *C*_max_ and delayed *t*_max_ after topical application of CsA gelatin microemulsion-based organogel in male SD rats and an AUC of 9.10 ± 1.28 μg. h/mL in rabbits after intravenous infusion of CsA-mixed micelles.^9,72^

There are reported advancements in IVIVC for oral formulations to guarantee the consistent performance of oral therapeutics.^73,74^ Unfortunately, there are currently no official guidelines or specific scientific reports available for the development of highly reliable *in vitro*–*in vivo* correlations for transdermal studies. In the study reported by Mohammed et al., the IVIVC was concluded based on the data obtained from the *in vitro* skin permeation studies of niacinamide and confocal Raman spectroscopy, with no experimental evidence from pharmacokinetic studies.^75^ Overall, the findings from this study offered additional validation for the utilization of confocal Raman spectroscopy to monitor the administration of drugs across and into the skin. However, based on the available reports, in our studies, the *in vivo* absorption data from the pharmacokinetic studies were utilized to understand and plot the graph against *in vitro* skin permeation studies of CsA-CAGE-P gel formulation to find out the correlation. There was an excellent point-to-point correlation (level A) between the *in vitro* % permeated across the porcine skin and *in vivo* % absorption in rats with *R*^2^ values of 0.991 and 0.992 for both linear and polynomial equations, respectively.

The efficacy studies were performed in the imiquimod-induced plaque psoriasis rat model. IMQ has been routinely used to induce psoriasis.^76,77^ The excess concentration of topically applied IMQ causes systemic inflammation of the spleen. The spleen plays an important role in the immune system and immune response, as it produces lymphocytes, monocytes, and plasma cells. The psoriasis inflammation causes enlargement of the spleen due to the huge increase in the number of cells, including Th17/Th22 T cells.^78,79^ Correlating with the previous studies, our work showed a significant enlargement of the spleen compared with the control group. Topical application of the CsA-CAGE-P gel showed a significant reduction in the splenomegaly, indicative of alleviation in the inflammatory response. Notably, the application of IMQ also causes keratinocytes and plasmacytoid dendritic cells to release pro-inflammatory cytokines. The increased levels of activated NF-κB, IL-12, and IL-23 activate circulating naïve T-helper lymphocytes which migrate to the skin site causing uncontrolled keratinocyte proliferation.^80,81^ The current treatment methods employed against psoriasis involve systemic administration of methotrexate, CsA, etanercept, and antibodies (ipilimumab).^82−85^ The best topical formulation, CsA-CAGE-P gel, was used for efficacy studies and compared with intravenous CsA administration. CsA inhibits the IL-2 receptor, which prevents the secondary production of pro-inflammatory T-cell cytokines and necrosis factors. IL-2-dependent immune amplification is suppressed sufficiently to keep cellular immune reactivity below a critical threshold.^86,87^ CsA was found to reduce the density of T-cell subsets in both the dermis and epidermis of psoriatic lesions.^88^ Attributed to the greater disposition of CsA within the psoriatic skin after CsA-CAGE-P gel application, the primary and secondary efficacy study objectives of reduced erythema, scaling, and epidermal thickness were achieved. Together, topical application of CsA in CAGE-P gel is a simple non-invasive and effective system to control plaque psoriasis.

## 5 Conclusion

The present work demonstrated the effectiveness of ionic liquid-based chemical enhancers in the skin transport of macromolecule, CsA. Free CsA did not permeate across the excised porcine skin. However, CsA permeated across the rat skin. The co-treatment of skin with CAGE ionic liquids enhanced CsA permeation significantly more than that of co-treatment with conventional fatty acid-based permeation enhancers, PA, and OA. Moreover, the effectiveness of CAGE in enhancing CsA skin permeation further increased after incorporation in Pluronic F127 gel. The biophysical studies showed an alteration of the skin barrier property after the application of CAGE. A clinically relevant plasma concentration of CsA was achieved after the topical application of the CAGE-Pluronic F127 gel. Notably, IVIVC showed a positive correlation, and the effectiveness of the fabricated CsA-CAGE-P gel formulation can be translated for further investigations in a clinical context. Taken together, CsA applied with CAGE-Pluronic F127 gel was efficacious in treating imiquimod-induced plaque psoriasis.

## Supporting Information Available

The supplementary tables (S1 and S2) and figures (S1, S2, S3, S4, S5, S6, S7, S8, S9, and S10) are mentioned in the additional supplementary file (PDF)
