# Supplementary material for: Ionic Liquid-Mediated Transdermal Delivery of Organogel Containing Cyclosporine A for the Effective Treatment of Psoriasis
Source: ACS Omega. 2024 Sep 25;9(40):41565–82. doi: 10.1021/acsomega.4c05346 (PMC11465456; doi:10.1021/acsomega.4c05346)
Supplement: Supplementary file 1 — ao4c05346_si_001.pdf [file ao4c05346_si_001.pdf]

Supplementary Material

Ionic liquid-mediated transdermal delivery of organogel containing cyclosporine A for the effective treatment of psoriasis

Deepanjan Datta[1,2]*, Sony Priyanka Bandi[1,3], Venkata Vamsi Krishna Venuganti[1]*

[1]Department of Pharmacy, Birla Institute of Technology and Science (BITS) Pilani, Hyderabad Campus, Hyderabad 500078, Telangana State, India

[2]Department of Pharmaceutics, Manipal College of Pharmaceutical Sciences, Manipal Academy of Higher Education, Manipal 576104, Karnataka State, India

[3]Loka Laboratories Private Limited, Technology Business Incubator, BITS Pilani Hyderabad Campus, Jawahar Nagar, Medchal 500078, Telangana, India

***Corresponding author**

Venkata Vamsi Krishna Venuganti, PhD
Professor and Dean
Department of Pharmacy, Birla Institute of Technology and Science (BITS) Pilani,
Hyderabad Campus, Hyderabad 500078, Telangana State.
Email: vamsi@hyderabad.bits-pilani.ac.in
Ph: +91-4066303581

Deepanjan Datta, PhD
Assistant Professor
Department of Pharmaceutics
Manipal College of Pharmaceutical Sciences
Manipal Academy of Higher Education, Manipal 576104, Karnataka State, India
Email: deepanjandtt@gmail.com; deepanjan.datta@manipal.edu
Ph: +91-9102514454

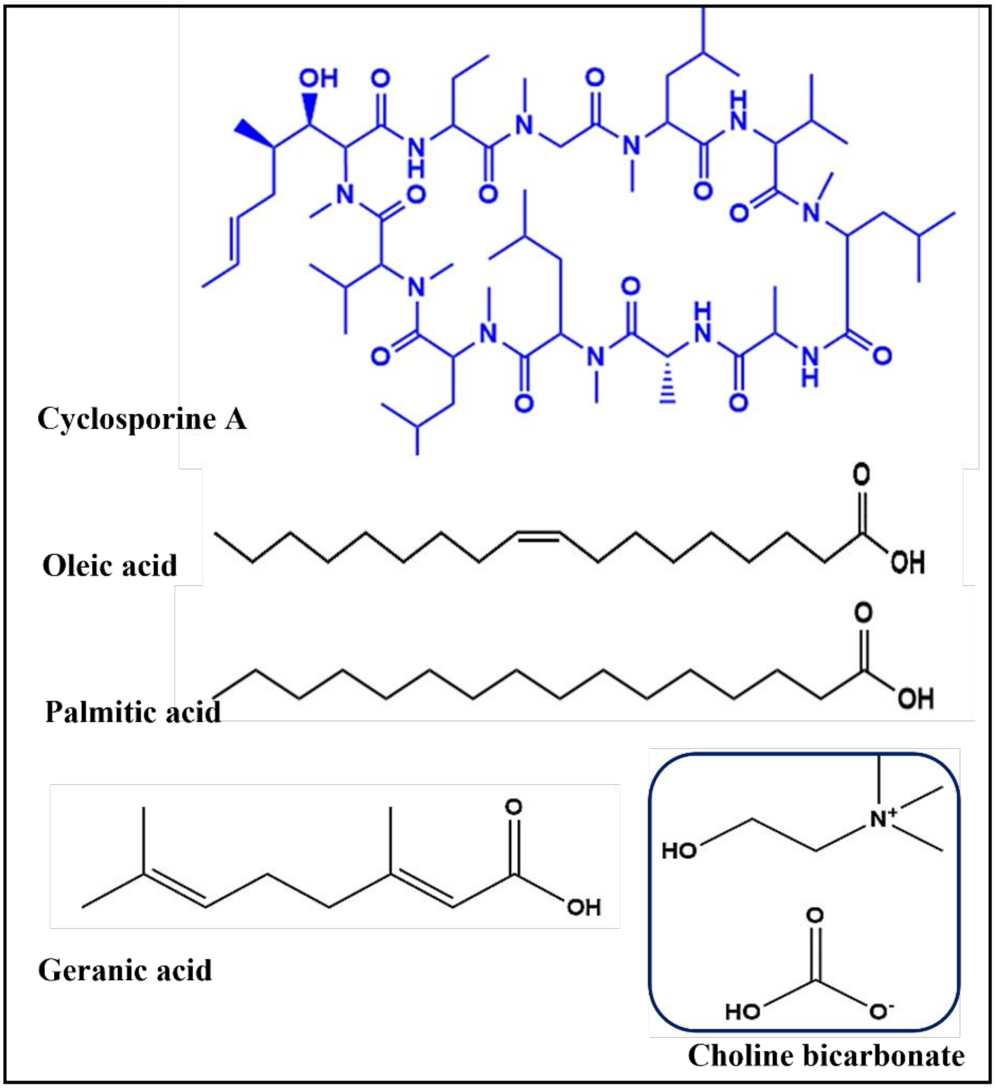

Supplementary Figure S1. Chemical structure of cyclosporine A, oleic acid, palmitic acid, geranic acid and choline bicarbonate.

Supplementary Table S1. Solubility of CsA in the presence of chemical permeation enhancers in different vehicles.

**Solvent**	**CsA: permeation enhancer ratio**	**Observation**
**CAGE**	1: [1:2 M]	Clear, yellowish, soluble
**Palmitic acid (PA)**		
IPA	1:0.5	soluble
PBS + IPA	1:0.4	precipitate
PBS + IPA	1: 0.25	soluble
**Oleic acid (OA)**		
IPA	1:0.5	soluble
PBS + IPA	1:0.4	turbid
PBS + IPA	1: 0.25	soluble

CsA – cyclosporine A; **CAGE** – choline bicarbonate and geranic acid ionic liquids; **PBS** - phosphate buffer saline (pH- 7.4); **IPA** - isopropyl alcohol. The solubility profile of CAGE or CPEs in combination with CsA (1%) was determined in 1 ml of the PBS-IPA mixture (65: 35%).

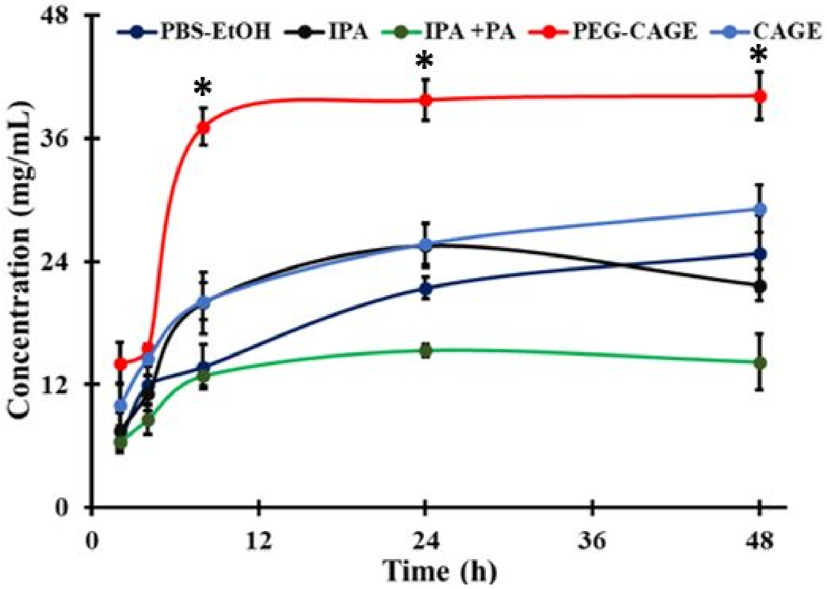

Supplementary Figure S2. Solubility profile of cyclosporine A in different vehicles after 48 h. The asterisk **(*)** represents that the value is significantly different at $p < 0.05$ compared to other groups. **PBS** – phosphate-buffered saline (pH 7.4); **IPA** – isopropyl alcohol; **PA** – palmitic acid**; PEG** – polyethylene glycol 400; **CAGE** – choline bicarbonate and geranic acid ionic liquids.

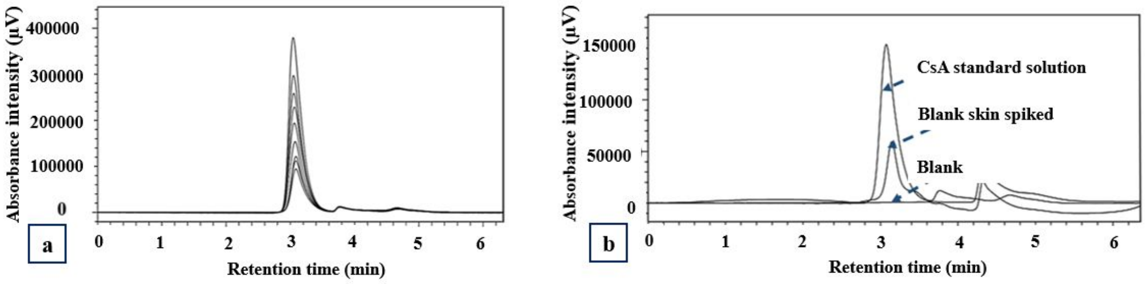

Supplementary Figure S3. RP-HPLC chromatogram of free CsA (0.5 - 100 µg/ml) in PBS-methanol (65: 35%) **(a)** and CsA calibration standard (20 µg/ml), blank skin spiked with CsA standard **(b)**.

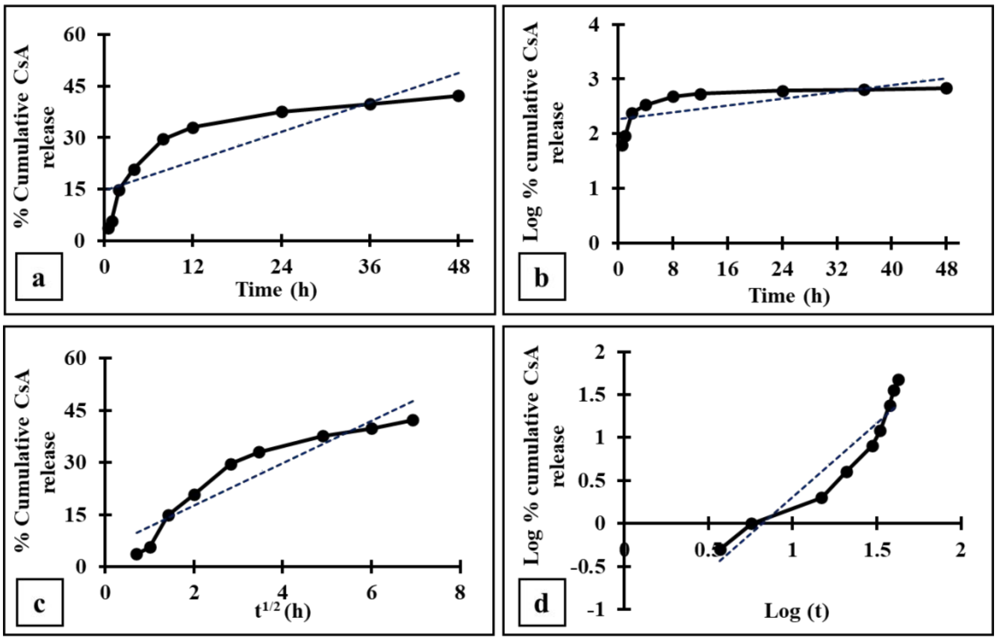

Supplementary Figure S4. Mathematical models for CsA release kinetics from CAGE-P gel formulation. Zero-order model (a); First order model (b); Higuchi model (c), and Korsmeyer-Peppas model (d). The correlation coefficients along with other parameters for each model for the release of CsA from CAGE-P gel are presented in the table below.

Supplementary Table S2. Release kinetics of CsA from CAGE-P gel formulation.

**Kinetic models**	**R^2 value**	**n**
Zero-order	0.71	-
First-order	0.49	-
Higuchi	0.87	-
Korsmeyer-Peppas (Power law)	0.90	1.7 (Super case II transport)

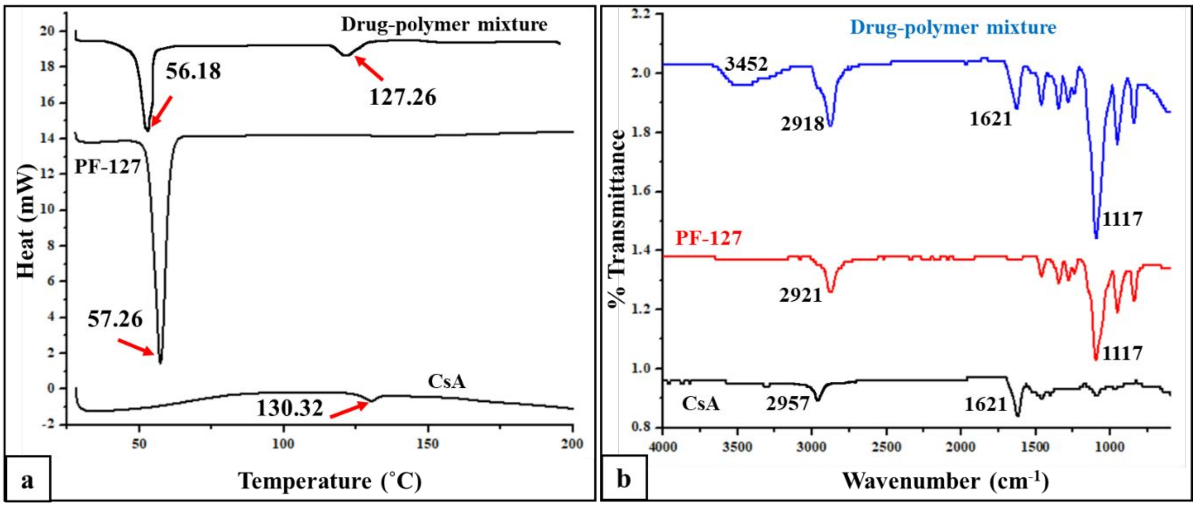

Supplementary Figure S5. Representative DSC thermograms (a) and FTIR spectra (b) of CsA, PF-127 and CsA+ PF-127 (drug-polymer mixture).

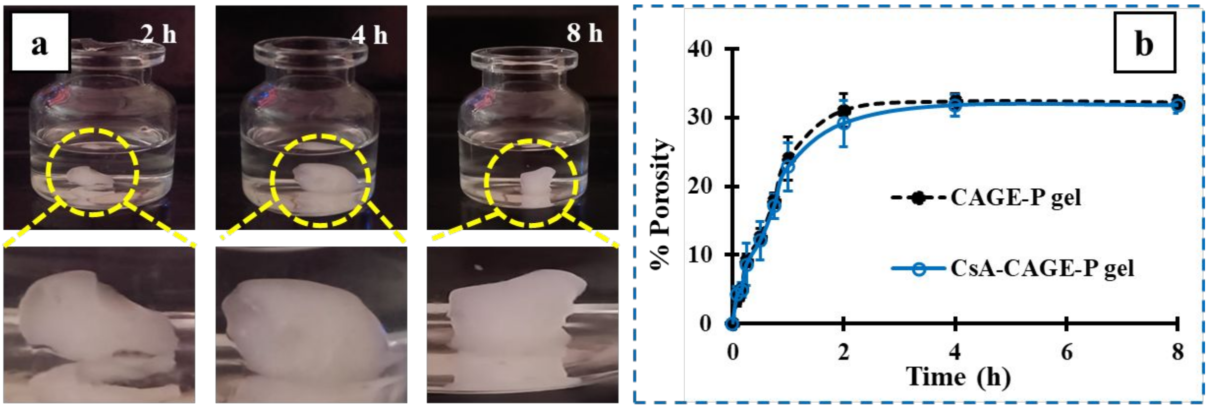

Supplementary Figure S6. Digital images were captured for the CsA-loaded CAGE-P gel formulation in absolute ethanol to perform porosity studies. The macroscopic images are shown to reveal the morphological changes at various time intervals **(a)**. The graph shows an increase in the porosity with time for both CsA-CAGE-P and CAGE-P gel formulation **(b)**.

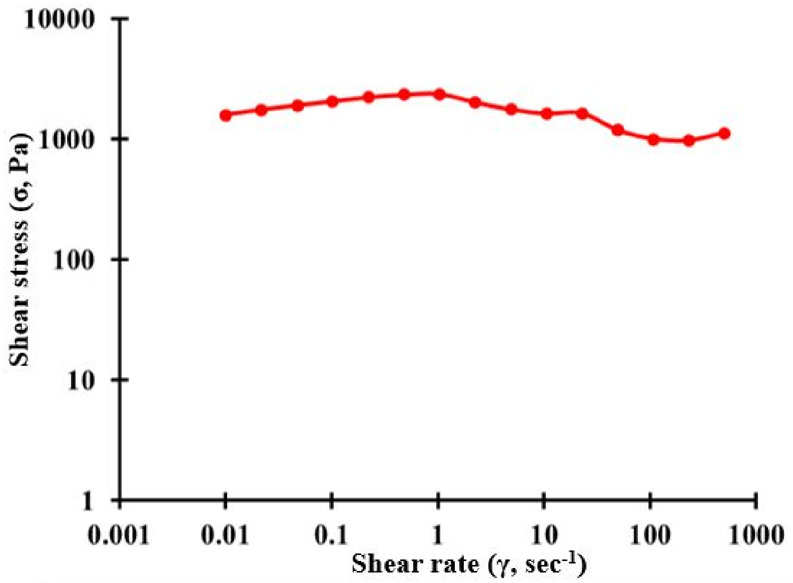

Formulation	K (Pa.s^n)	n
CsA-CAGE-P gel	1734.5	- 0.06

Supplementary Figure S7. Graph showing the shear stress values with varying shear rates for CsA- loaded CAGE Pluronic (CsA-CAGE-P) gel formulation with consistency factor (K; Pa.s^n) and shear thinning flow behaviour ($n < 1$) at 25°C. Values are represented as Mean ± SD (n = 4).

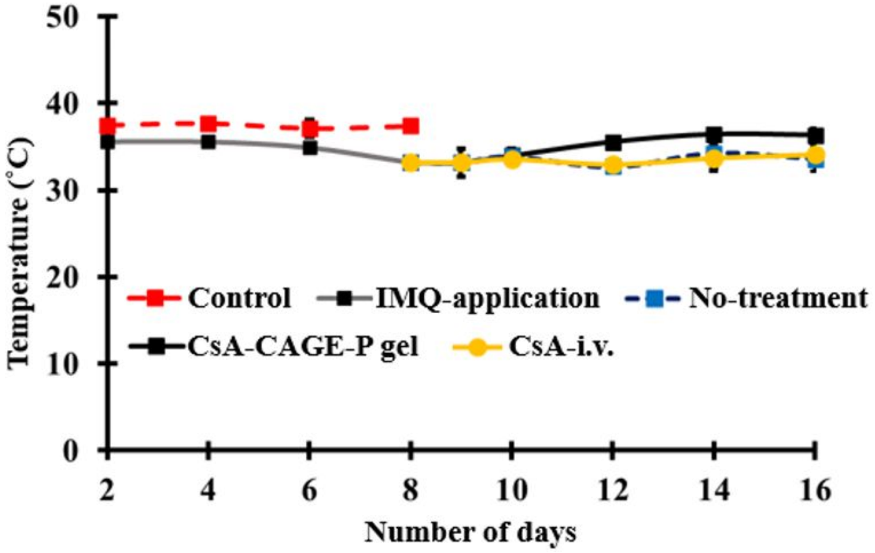

Supplementary Figure S8. Change in body temperature of *Sprague Dawley* rats treated with different formulations for 8 consecutive days. Data represent mean ± SD (n =4).

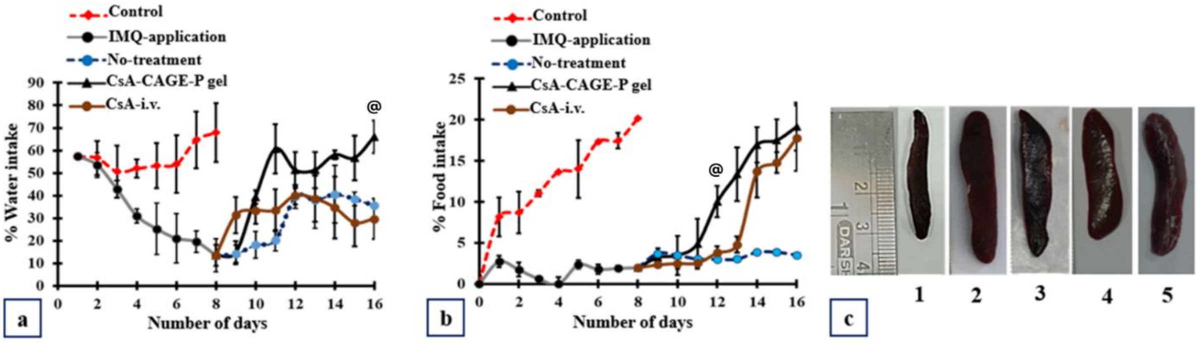

Supplementary Figure S9. The percentage of water intake **(a)** and food intake **(b)** by rats induced with psoriasis and treated with different formulations. The excised spleen was harvested after sacrificing the rats after treatment (**c**). Control **(1)**, IMQ-application **(2)**; No-treatment **(3)**; CsA-CAGE-P gel **(4)** and CsA-i.v. **(5)**. **"@"** represents that the value is significantly different at $p < 0.05$ compared to other groups, except the control.

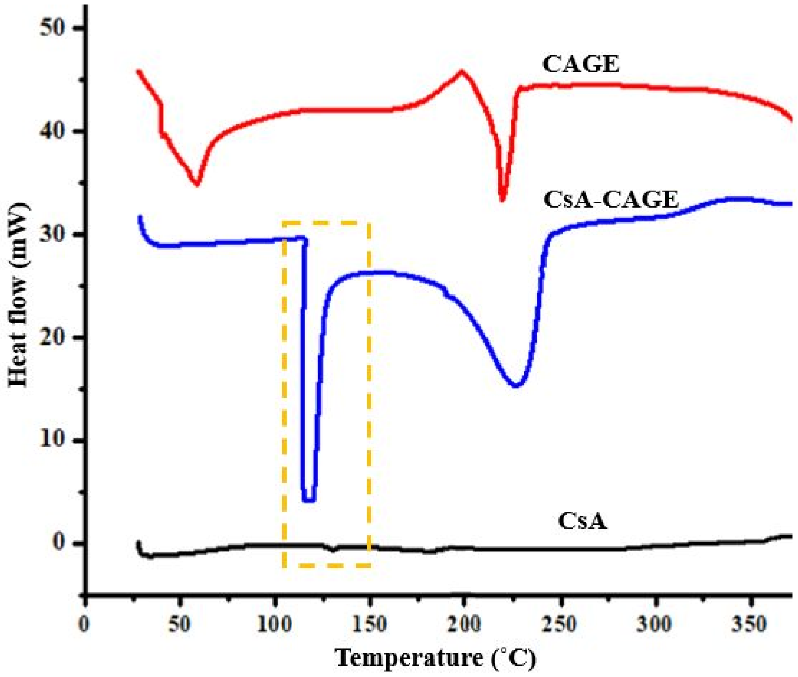

Supplementary Figure S10. Representative DSC thermograms of CsA, CsA-CAGE and CAGE.
